# Studies of Metabolic Phenotypic Correlates of 15 Obesity Associated Gene Variants

**DOI:** 10.1371/journal.pone.0023531

**Published:** 2011-09-02

**Authors:** Camilla Helene Sandholt, Marie Aare Vestmar, Dorthe Sadowa Bille, Anders Borglykke, Katrine Almind, Lars Hansen, Annelli Sandbæk, Torsten Lauritzen, Daniel Witte, Torben Jørgensen, Oluf Pedersen, Torben Hansen

**Affiliations:** 1 The Novo Nordisk Foundation Center for Basic Metabolic Research, Section of Metabolic Genetics Faculty of Health Sciences, University of Copenhagen, Copenhagen, Denmark; 2 Novo Nordisk A/S, Biomarkers, Inflammation & GH, Global Development, Bagsværd, Denmark; 3 Faculty of Health Sciences, University of Copenhagen, Copenhagen, Denmark; 4 Department of Paediatrics, Holbæk Hospital, Holbæk, Denmark; 5 Research Centre for Prevention and Health, Glostrup University Hospital, Glostrup, Denmark; 6 Bristol-Meyers Squibb & Co., Discovery Medicine and Clinical Pharmacology, CV Metabolic Disease, Princeton, New Jersey, United States of America; 7 Department of General Practice, University of Aarhus, Aarhus, Denmark; 8 Steno Diabetes Center, Gentofte, Denmark; 9 Faculty of Health Science, University of Aarhus, Aarhus, Denmark; 10 Faculty of Health Sciences, University of Southern Denmark, Odense, Denmark; German Diabetes Center, Leibniz Center for Diabetes Research at Heinrich Heine University, Germany

## Abstract

**Aims:**

Genome-wide association studies have identified novel BMI/obesity associated susceptibility loci. The purpose of this study is to determine associations with overweight, obesity, morbid obesity and/or general adiposity in a Danish population. Moreover, we want to investigate if these loci associate with type 2 diabetes and to elucidate potential underlying metabolic mechanisms.

**Methods:**

15 gene variants in 14 loci including *TMEM18* (rs7561317), *SH2B1* (rs7498665), *KCTD15* (rs29941), *NEGR1* (rs2568958), *ETV5* (rs7647305), *BDNF* (rs4923461, rs925946), *SEC16B* (rs10913469), *FAIM2* (rs7138803), *GNPDA2* (rs10938397), *MTCH2* (rs10838738), *BAT2* (rs2260000), *NPC1* (rs1805081), *MAF* (rs1424233), and *PTER* (rs10508503) were genotyped in 18,014 middle-aged Danes.

**Results:**

Five of the 15 gene variants associated with overweight, obesity and/or morbid obesity. Per allele ORs ranged from 1.15–1.20 for overweight, 1.10–1.25 for obesity, and 1.41–1.46 for morbid obesity. Five of the 15 variants moreover associated with increased measures of adiposity. *BDNF* rs4923461 displayed a borderline BMI-dependent protective effect on type 2 diabetes (0.87 (0.78–0.96, *p* = 0.008)), whereas *SH2B1* rs7498665 associated with nominally BMI-independent increased risk of type 2 diabetes (1.16 (1.07–1.27, *p* = 7.8×10^−4^)).

**Conclusions:**

Associations with overweight and/or obesity and measures of obesity were confirmed for seven out of the 15 gene variants. The obesity risk allele of *BDNF* rs4923461 protected against type 2 diabetes, which could suggest neuronal and peripheral distinctive ways of actions for the protein. *SH2B1* rs7498665 associated with type 2 diabetes independently of BMI.

## Introduction

Several studies have established that a genetic contribution to the pathogenesis of obesity exists [1]–[3]. Since the prevalence of obesity is increasing at alarming rates worldwide, great efforts have been made to identify the genetic components predisposing some individuals to accumulate more body fat. Especially searches for disease-associated single nucleotide polymorphisms (SNPs) have been prioritised, but without noticeable success prior to 2007 where genome-wide association studies (GWAS) were introduced. Several GWAS of body mass index (BMI) and/or obesity have been performed, and the first wave resulted in the suggestion of SNPs in/near the *INSIG2* [4], *FTO* [5], *PFKP* [6], and *CTNNBL1* [7] genes, however, only SNPs in the *FTO* locus were convincingly replicated in other GWAS [6], [8] and independent replication studies [9]–[11]. At the end of the first obesity GWAS wave, SNPs near the *MC4R* gene were identified, both in an independent GWAS [12] and through meta-analysis of several GWAS [13]. These associations have subsequently been confirmed in independent replication studies [14], [15]. At the same time, another obesity gene, *PCSK1*, was suggested using re-sequencing of linkage peaks [16], and some replication studies have established week associations between this gene and obesity related measures [17], [18].

In 2009, the second wave of BMI/obesity GWAS were performed, identifying 15 novel susceptibility loci. A GWAS of BMI and weight in almost 32,000 individuals of primarily European descendant validated the associations with *FTO* and *MC4R*. Moreover, genome-wide significant associations for variants in/near two genes formerly suggested as biological candidate genes for obesity, *BDNF* and *SH2B1* were observed, together with the identification of seven new BMI/weight loci, *TMEM18*, *KCTD15*, *NEGR1*, *SEC16B*, *ETV5*, *FAIM2* and *BAT2* [19]. A meta-analysis of GWAS on BMI comprising more than 32,000 individuals also confirmed the associations of *FTO* and *MC4R*, and furthermore identified six new loci; four of them overlapping with the other BMI/weight GWAS; *SH2B1*, *TMEM18*, *KCTD15* and *NEGR1* and two novel loci *GNPDA2* and *MTCH2* [20]. The last GWAS of the second wave identified three novel loci, *NPC1*, *MAF* and *PTER* in individuals with early-onset morbid adult obesity [21].

Given the hypothesis-free approach used in GWAS no obvious candidacy could be explained for most of the suggested loci. However, a predominance of the genes nearest the identified SNPs were expressed in the brain, and several particularly in the hypothalamus, suggesting putative roles in the regulation of appetite and energy expenditure [19], [20], [22].

Since there in the first GWAS wave were identified six loci, but only three *FTO*, *MC4R* and *PCSK1* were confirmed in independent GWAS or replication studies, the importance of independent replication studies to distinguish between true associations and “winners curse” observations is underlined. Therefore, we attempt to confirm reported GWAS findings of association with obesity in Danish study samples. Furthermore, obesity is a strong risk factor of type 2 diabetes and other key metabolic traits, and therefore, the aim of the present study is also to elucidate whether 15 SNPs in/near the 14 loci from the second obesity GWAS wave associate with type 2 diabetes and other key metabolic correlates.

## Results

### Confirmation of associations with overweight and obesity

The *BDNF* rs4923461 A-allele associated with increased risk of overweight, with a per allele OR of 1.15 (1.07–1.24, *p* = 2.5×10^−4^), and borderline with obesity with a per allele OR of 1.14 (1.05–1.23, *p* = 0.002) but not with morbid obesity (Table 1, Table S4). This suggested that the effect of the allele might occur at a lower BMI, thus stratifying at 22.5 kg/m^2^ showed a higher effect size with an allelic OR of 1.21 (1.09–1.34, *p* = 4.0×10^−4^) (data not shown). Similarly, *BDNF* rs925946 associated with overweight, with an OR of 1.20 (1.12–1.29, *p* = 1.4×10^−7^), and obesity with an OR of 1.15 (1.06–1.24, *p* = 3.9×10^−4^) but not with morbid obesity. Accordingly, stratifying at 22.5 kg/m^2^ showed an OR of 1.27 (1.15–1.40, *p* = 1.8×10^−6^) per T-allele (data not shown).

**Table 1:** Case-control studies of overweight, obesity and type 2 diabetes in the combined study sample.

				Obesity			Type 2 diabetes	
SNP	Locus	Risk allele	RAF (%)	OR_overweight_	OR_obesity_	OR_morbid obesity_	OR _Without BMI adjustment_	*OR _With BMI adjustment_
rs7561317	*TMEM18*	G	84	1.08	1.25	1.46	1.13	1.04
				(0.99–1.17)	(1.14–1.37)	(1.17–1.82)	(1.02–1.26)	(0.92–1.17)
				*p* = 0.08	*p* = 2.1×10^−6^	*p* = 8.3×10^−4^	*p* = 0.02	*p* = 0.52
rs7498665	*SH2B1*	G	42	1.01	1.06	1.17	1.18	1.16
				(0.95–1.08)	(0.99–1.13)	(1.00–1.36)	(1.09–1.28)	(1.07–1.27)
				*p* = 0.74	*p* = 0.12	*p* = 0.05	*p* = 3.0×10^−5^	*p* = 7.8×10^−4^
rs29941	*KCTD15*	G	68	1.00	1.02	1.09	0.98	0.99
				(0.94–1.07)	(0.95–1.10)	(0.93–1.28)	(0.90–1.07)	(0.90–1.09)
				*p* = 0.93	*p* = 0.60	*p* = 0.30	*p* = 0.64	*p* = 0.79
rs2568958	*NEGR1*	A	60	1.01	1.04	1.09	1.08	1.06
				(0.95–1.08)	(0.97–1.11)	(0.93–1.27)	(1.00–1.17)	(0.97–1.16)
				*p* = 0.75	*p* = 0.28	*p* = 0.28	*p* = 0.05	*p* = 0.2
rs7647305	*ETV5*	C	81	1.10	1.18	1.41	1.12	1.04
				(1.02–1.19)	(1.08–1.29)	(1.15–1.74)	(1.02–1.24)	(0.93–1.16)
				*p* = 0.02	*p* = 1.8×10^−4^	*p* = 0.001	*p* = 0.02	*p* = 0.5
rs4923461	*BDNF*	A	78	1.15	1.14	1.12	0.96	0.87
				(1.07–1.24)	(1.05–1.23)	(0.94–1.35)	(0.87–1.05)	(0.78–0.96)
				*p* = 2.5×10^−4^	*p* = 0.002	*p* = 0.21	*p* = 0.4	*p* = 0.008
rs925946	*BDNF*	T	31	1.20	1.15	1.16	0.97	0.91
				(1.12–1.29)	(1.06–1.24)	(0.98–1.37)	(0.89–1.06)	(0.83–1.00)
				*p* = 1.4×10^−7^	*p* = 3.9×10^−4^	*p* = 0.08	*p* = 0.5	*p* = 0.06
rs10913469	*SEC16B*	C	21	1.04	1.11	1.11	0.97	0.90
				(0.96–1.12)	(1.03–1.21)	(0.93–1.34)	(0.88–1.06)	(0.81–1.00)
				*p* = 0.32	*p* = 0.01	*p* = 0.25	*p* = 0.47	*p* = 0.05
rs7138803	*FAIM2*	A	41	1.03	1.09	1.19	1.08	1.06
				(0.97–1.10)	(1.02–1.17)	(1.02–1.38)	(1.00–1.17)	(0.97–1.15)
				*p* = 0.33	*p* = 0.01	*p* = 0.03	*p* = 0.06	*p* = 0.23
rs10938397	*GNPDA2*	G	41	1.09	1.15	1.20	1.06	0.99
				(1.02–1.16)	(1.07–1.23)	(1.03–1.4)	(0.97–1.14)	(0.90–1.08)
				*p* = 0.01	*p* = 1.1×10^−4^	*p* = 0.02	*p* = 0.19	*p* = 0.79
rs10838738	*MTCH2*	G	35	1.00	1.01	0.99	1.04	1.03
				(0.94–1.07)	(0.94–1.08)	(0.85–1.17)	(0.96–1.13)	(0.94–1.13)
				*p* = 0.91	*p* = 0.86	*p* = 0.94	*p* = 0.34	*p* = 0.49
rs2260000	*BAT2*	T	64	1.06	1.01	1.05	0.93	0.91
				(0.99–1.14)	(0.94–1.09)	(0.90–1.24)	(0.85–1.01)	(0.83–1.00)
				*p* = 0.10	*p* = 0.81	*p* = 0.51	*p* = 0.08	*p* = 0.05
rs1805081	*NPC1*	A	58	1.06	1.10	1.21	1.09	1.07
				(1.00–1.13)	(1.03–1.18)	(1.03–1.41)	(1.01–1.18)	(0.98–1.17)
				*p* = 0.07	*p* = 0.005	*p* = 0.02	*p* = 0.03	*p* = 0.13
rs1424233	*MAF*	G	47	1.05	1.06	1.07	0.97	0.94
				(0.99–1.12)	(0.99–1.14)	(0.92–1.25)	(0.89–1.05)	(0.86–1.02)
				*p* = 0.11	*p* = 0.08	*p* = 0.36	*p* = 0.40	*p* = 0.14
rs10508503	*PTER*	T	8	0.94	0.99	1.07	0.93	0.91
				(0.84–1.05)	(0.87–1.12)	(0.82–1.40)	(0.81–1.07)	(0.78–1.07)
				*p* = 0.29	*p* = 0.84	*p* = 0.61	*p* = 0.33	*p* = 0.26

Data are presented as odds ratios (OR) (CI 95%) and *p*-values. OR and *p*-values are specified for the additive model and adjusted for age and gender.

*Association with type 2 diabetes are additionally adjusted for BMI. RAF; risk allele frequency.

A different pattern was observed for the *TMEM18* rs7561317 G-allele, which strongly associated with obesity with an OR of 1.25 (1.14–1.37, *p* = 2.1×10^−6^) and with morbid obesity with an OR of 1.46 (1.17–1.82, *p* = 8.3×10^−4^) per allele, but not with overweight.

Both the *ETV5* rs7647305 C-allele and the *GNPDA2* rs10938397 A-allele associated with increased risk of obesity with ORs 1.18 (1.08–1.29, *p* = 1.8×10^−4^) (rs7647305) and 1.15 (1.07–1.23, *p* = 1.1×10^−4^)) (rs10938397), but not with overweight or morbid obesity.

### Association with type 2 diabetes

Only the *SH2B1* rs7498665 G-allele strongly associated with increased risk of type 2 diabetes when adjusting for age and sex with an allelic OR of 1.18 (1.09–1.28, *p* = 3.0×10^−5^) and when additionally adjusting for BMI a nominal association sustained with an OR of 1.16 (1.07–1.27, *p* = 7.8×10^−4^).

The *BDNF* rs4923461 A-allele showed a tendency towards a reduced risk of type 2 diabetes, however, the association was merely nominally, with an OR of 0.87 (0.78–0.96, *p* = 0.008) per allele when adjusting for BMI (Table 1, Table S4), however, when omitting adjustments for BMI the nominally tendency disappeared. The contradictory risk altering patterns of obesity and type 2 diabetes could suggest an interaction between the risk allele and BMI, however, no such interaction was found (data not shown).

### Associations with anthropometric measures

The *BDNF* rs925946 T-allele associated significantly/borderline with three increased measures of adiposity, BMI: 0.35 kg/m^2^ (0.18–0.53, *p* = 8.2×10^−5^), waist circumference: 0.76 cm (0.31–1.21, *p* = 8.7×10^−4^), weight 1.05 kg (0.49–1.60, *p* = 2.4×10^−4^) whereas the rs4923461 A-allele only associated nominally (Table 2, Table S5). The highest per allele effect sizes of measures of adiposity were observed for the *TMEM18* rs7561317 G-allele with increases in BMI of 0.52 kg/m^2^ (0.30–0.74, *p* = 2.9×10^−6^), in waist circumference of 1.05 cm (0.50–1.61, *p* = 1.8×10^−4^) and in weight of 1.46 kg (0.77–2.15, *p* = 3.1×10^−5^). Although the A-allele of *FAIM2* rs7138803 did not reach statistical significance in the case-control analyses of overweight and obesity, this risk allele strongly associated with increased measures of adiposity with per allele effects on BMI of 0.41 kg/m^2^ (0.25–0.57, *p* = 8.2×10^−7^), on waist circumference of 1.06 cm (0.65–1.48, *p* = 4.9×10^−7^) and on weight of 1.41 kg (0.90–1.93, *p* = 7.6×10^−8^) (Table 2, Table S5). *GNPDA2* rs10938397 and *SEC16B* rs10913469 both associated nominally with increased BMI and waist circumference (Table 2, Table S5).

**Table 2:** Effects of and associations with the obesity risk alleles on measures of adiposity in the population-based Inter99 cohort.

				Measures of adiposity			
SNP	Locus	Risk allele	RAF (%)	BMI (kg/m^2^)	Waist circumference (cm)	Weight (kg)	Waist-to-hip ratio
rs7561317	*TMEM18*	G	83	0.52	1.05	1.46	0.003
				(0.30–0.74)	(0.50–1.61)	(0.77–2.15)	(0.0002–0.006)
				*p* = 2.9×10^−6^	*p* = 1.8×10^−4^	*p* = 3.1×10^−5^	*p* = 0.04
rs7498665	*SH2B1*	G	41	0.08	0.23	0.24	0.001
				(−0.09–0.24)	(−0.19–0.64)	(−0.25–0.78)	(−0.001–0.003)
				*p* = 0.35	*p* = 0.28	*p* = 0.31	*p* = 0.28
rs29941	*KCTD15*	G	68	0.16	0.29	0.53	0.0008
				(−0.02–0.33)	(−0.14–0.73)	(−0.01–1.07)	(−0.002–0.003)
				*p* = 0.08	*p* = 0.19	*p* = 0.06	*p* = 0.49
rs2568958	*NEGR1*	A	59	0.06	0.17	0.20	0.002
				(−0.10–0.22)	(−0.24–0.58)	(−0.31–0.72)	(−0.0003–0.004)
				*p* = 0.46	*p* = 0.42	*p* = 0.44	*p* = 0.09
rs7647305	*ETV5*	C	80	0.22	0.70	0.69	0.003
				(0.01–0.42)	(0.18–1.22)	(0.04–1.34)	(0.0005–0.006)
				*p* = 0.04	*p* = 0.008	*p* = 0.04	*p* = 0.02
rs4923461	*BDNF*	A	78	0.29	0.61	0.89	0.0007
				(0.10–0.49)	(0.11–1.11)	(0.26–1.51)	(−0.002–0.003)
				*p* = 0.004	*p* = 0.02	*p* = 0.005	*p* = 0.63
rs925946	*BDNF*	T	31	0.35	0.76	1.05	0.001
				(0.18–0.53)	(0.31–1.21)	(0.49–1.60)	(−0.0009–0.004)
				*p* = 8.2×10^−5^	*p* = 8.7×10^−4^	*p* = 2.4×10^−4^	*p* = 0.23
rs10913469	*SEC16B*	C	21	0.33	0.83	0.90	0.003
				(0.14–0.53)	(0.33-1-33)	(0.27–1.52)	(0.00004–0.005)
				*p* = 9.2×10^−4^	*p* = 0.001	*p* = 0.005	*p* = 0.05
rs7138803	*FAIM2*	A	40	0.41	1.06	1.41	0.003
				(0.25–0.57)	(0.65–1.48)	(0.90–1.93)	(0.001–0.005)
				*p* = 8.2×10^−7^	*p* = 4.9×10^−7^	*p* = 7.6×10^−8^	*p* = 0.005
rs10938397	*GNPDA2*	G	41	0.28	0.61	0.73	0.002
				(0.11–0.44)	(0.19–1.03)	(0.21–1.26)	(−0.0003–0.004)
				*p* = 0.001	*p* = 0.004	*p* = 0.006	*p* = 0.1
rs10838738	*MTCH2*	G	35	−0.04	0.007	−0.12	−0.0004
				(−0.21–0.13)	(−0.43–0.44)	(−0.66–0.42)	(−0.003–0.002)
				*p* = 0.66	*p* = 0.97	*p* = 0.66	*p* = 0.74
rs2260000	*BAT2*	T	63	0.07	0.29	0.58	0.0004
				(−0.09–0.24)	(−0.14–0.71)	(0.05–1.10)	(−0.002–0.003)
				*p* = 0.39	*p* = 0.18	*p* = 0.03	*p* = 0.74
rs1805081	*NPC1*	A	57	0.11	0.25	0.19	0.002
				(−0.05–0.28)	(−0.17–0.66)	(−0.33–0.71)	(−0.0002–0.004)
				*p* = 0.18	*p* = 0.25	*p* = 0.48	*p* = 0.08
rs1424233	*MAF*	G	47	0.15	0.16	0.44	−0.0004
				(−0.01–0.31)	(−0.25–0.58)	(−0.07–0.95)	(−0.003–0.002)
				*p* = 0.07	*p* = 0.44	*p* = 0.09	*p* = 0.72
rs10508503	*PTER*	T	9	0.07	0.06	0.33	−0.002
				(−0.22–0.36)	(−0.68–0.79)	(−0.58–1.24)	(−0.006–0.002)
				*p* = 0.63	*p* = 0.88	*p* = 0.48	*p* = 0.4

Data are presented as effect sizes (CI 95%) per allele and *p*-values. Effect sizes are given in absolute values and *p*-values are specified for the additive model and adjusted for age and gender. RAF; risk allele frequency.

### Associations with metabolic traits

*BDNF* rs925949 associated borderline with elevated levels of circulating leptin, with a 6% (3–10%, *p* = 8.3×10^−4^) per allele increase (ng/ml), unadjusted for BMI (Table 3 (data only shown for BMI adjusted effect sizes), Table S5). Furthermore, this risk allele displayed a tendency towards increased levels of fasting plasma glucose levels, however, associations were merely nominally (Table 4, Table S5). Nominally associations were also observed for the *FAIM2* rs7138803 A-allele which with elevated levels of circulating leptin as well as lower levels of HDL-cholesterol (Table 3, Table S5).

**Table 3:** Effects sizes of fasting serum lipids, adipokines and CRP, with and without adjustments for BMI in the population-based Inter99 cohort.

		Fasting serum lipids (mmol/l)				Fasting serum adipokines and CRP (ng/ml)		
SNP	Locus	Triglycerides	Total cholesterol	HDL-cholesterol	LDL-cholesterol	Leptin	Adiponectin	CRP
rs7561317	*TMEM18*	−0.005	−0.02	0.001	−0.04	−0.003	−0.0004	0.03
		(−0.03–0.02)	(−0.07–0.03)	(−0.02–0.02)	(−0.1–0.02)	(−0.04–0.03)	(−0.04–0.04)	(−0.03–0.09)
		a *p* = 0.67	a *p* = 0.51	a *p* = 0.87	a *p* = 0.21	a *p* = 0.87	a *p* = 0.99	a *p* = 0.37
		*p* = 0.23	*p* = 1.0	*p* = 0.16	*p* = 0.58	*p* = 0.01	*p* = 0.41	*p* = 0.02
rs7498665	*SH2B1*	−0.01	−0.01	0.008	−0.01	−0.01	−0.02	−0.04
		(−0.03–0.005)	(−0.05–0.02)	(−0.005–0.02)	(−0.06–0.03)	(−0.04–0.02)	(−0.05–0.004)	(−0.08–0.01)
		a *p* = 0.17	a *p* = 0.45	a *p* = 0.22	a *p* = 0.67	a *p* = 0.45	a *p* = 0.09	a *p* = 0.12
		*p* = 0.32	*p* = 0.54	*p* = 0.39	*p* = 0.59	*p* = 0.7	*p* = 0.09	*p* = 0.15
rs29941	*KCTD15*	0.009	0.02	0.00008	0.002	0.001	0.0005	−0.02
		(−0.01–0.03)	(−0.01–0.06)	(−0.01–0.01)	(−0.03–0.07)	(−0.03–0.03)	(−0.03–0.03)	(−0.07–0.02)
		a *p* = 0.37	a *p* = 0.20	a *p* = 0.99	a *p* = 0.50	a *p* = 0.94	a *p* = 0.97	a *p* = 0.31
		*p* = 0.15	*p* = 0.13	*p* = 0.58	*p* = 0.34	*p* = 0.37	*p* = 0.82	*p* = 0.79
rs2568958	*NEGR1*	−0.01	−0.03	0.01	−0.06	0.01	0.005	0.001
		(−0.03–0.005)	(−0.07–0.008)	(−0.0003–0.03)	(−0.1–(−0.02))	(−0.02–0.04)	(−0.02–0.03)	(−0.04–0.05)
		a *p* = 0.16	a *p* = 0.13	a *p* = 0.06	a *p* = 0.01	a *p* = 0.40	a *p* = 0.75	a *p* = 0.96
		*p* = 0.29	*p* = 0.16	*p* = 0.12	*p* = 0.009	*p* = 0.24	*p* = 0.86	*p* = 0.84
rs7647305	*ETV5*	0.01	0.03	0.008	−0.03	0.01	0.01	−0.02
		(−0.012–0.03)	(−0.02–0.08)	(−0.008–0.02)	(−0.09–0.03)	(−0.02–0.05)	(−0.02–0.04)	(−0.08–0.04)
		a *p* = 0.40	a *p* = 0.20	a *p* = 0.35	a *p* = 0.31	a *p* = 0.49	a *p* = 0.46	a *p* = 0.52
		*p* = 0.14	*p* = 0.12	*p* = 0.81	*p* = 0.42	*p* = 0.05	*p* = 0.76	*p* = 0.82
rs4923461	*BDNF*	−0.01	−0.01	0.005	−0.03	−0.008	0.006	0.02
		(−0.03–0.01)	(−0.06–0.03)	(−0.01–0.02)	(−0.09–0.03)	(−0.04–0.03)	(−0.03–0.04)	(−0.04–0.08)
		a *p* = 0.34	a *p* = 0.57	a *p* = 0.54	a *p* = 0.28	a *p* = 0.65	a *p* = 0.73	a *p* = 0.48
		*p* = 0.92	*p* = 0.88	*p* = 0.68	*p* = 0.39	*p* = 0.22	*p* = 0.92	*p* = 0.13
rs925946	*BDNF*	−0.02	−0.01	0.009	−0.01	0.02	0.02	0.04
		(−0.04-(−0.0009))	(−0.05–0.03)	(−0.005–0.02)	(−0.07–0.04)	(−0.01–0.05)	(−0.01–0.05)	(−0.01–0.09)
		a *p* = 0.04	a *p* = 0.53	a *p* = 0.21	a *p* = 0.60	a *p* = 0.24	a *p* = 0.18	a *p* = 0.13
		*p* = 0.55	*p* = 0.93	*p* = 0.93	*p* = 0.90	*p* = 0.0008	*p* = 0.52	*p* = 0.006
rs10913469	*SEC16B*	0.008	0.02	0.004	−0.02	−0.03	0.02	0.01
		(−0.01–0.03)	(−0.03–0.06)	(−0.01–0.02)	(−0.08–0.05)	(−0.06–0.006)	(−0.02–0.05)	(−0.04–0.07)
		a *p* = 0.49	a *p* = 0.42	a *p* = 0.61	a *p* = 0.62	a *p* = 0.11	a *p* = 0.36	a *p* = 0.70
		*p* = 0.07	*p* = 0.21	*p* = 0.52	*p* = 0.99	*p* = 0.52	*p* = 0.75	*p* = 0.09
rs7138803	*FAIM2*	−0.0001	−0.01	−0.01	−0.01	0.006	0.01	−0.03
		(−0.02–0.02)	(−0.05–0.03)	(−0.02–0.003)	(−0.06–0.04)	(−0.02–0.03)	(−0.04–0.02)	(−0.08–0.01)
		a *p* = 0.99	a *p* = 0.56	a *p* = 0.13	a *p* = 0.71	a *p* = 0.65	a *p* = 0.37	a *p* = 0.18
		*p* = 0.10	*p* = 0.91	*p* = 0.003	*p* = 0.61	*p* = 0.001	*p* = 0.08	*p* = 0.66
rs10938397	*GNPDA2*	−0.01	0.03	0.008	0.02	−0.005	0.01	0.02
		(−0.03–0.007)	(−0.01–0.06)	(−0.005–0.02)	(−0.03–0.07)	(−0.03–0.02)	(−0.02–0.04)	(−0.03–0.06)
		a *p* = 0.24	a *p* = 0.17	a *p* = 0.22	a *p* = 0.35	a *p* = 0.71	a *p* = 0.37	a *p* = 0.48
		*p* = 0.98	*p* = 0.07	*p* = 0.96	*p* = 0.20	*p* = 0.07	*p* = 0.79	*p* = 0.06
rs10838738	*MTCH2*	−0.002	−0.02	−0.02	−0.02	0.02	0.01	−0.0007
		(−0.02–0.02)	(−0.06–0.02)	(−0.04-(−0.008))	(−0.07–0.04)	(−0.007–0.05)	(−0.02–0.04)	(−0.05–0.05)
		a *p* = 0.83	a *p* = 0.36	a *p* = 0.002	a *p* = 0.54	a *p* = 0.14	a *p* = 0.47	a *p* = 0.98
		*p* = 0.71	*p* = 0.33	*p* = 0.006	*p* = 0.59	*p* = 0.48	*p* = 0.40	*p* = 0.83
rs2260000	*BAT2*	0.02	0.007	−0.01	0.02	−0.005	−0.0005	−0.02
		(−0.002–0.03)	(−0.03–0.04)	(−0.03-(−0.0005))	(−0.03–0.07)	(−0.03–0.02)	(−0.03–0.03)	(−0.07–0.03)
		a *p* = 0.08	a *p* = 0.71	a *p* = 0.04	a *p* = 0.39	a *p* = 0.70	a *p* = 0.98	a *p* = 0.40
		*p* = 0.05	*p* = 0.63	*p* = 0.03	*p* = 0.31	*p* = 0.84	*p* = 0.88	*p* = 0.48
rs1805081	*NPC1*	0.01	0.04	−0.0005	−0.0008	0.0003	−0.009	−0.03
		(−0.007–0.03)	(0.006–0.08)	(−0.01–0.01)	(−0.05–0.05)	(−0.03–0.03)	(−0.04–0.02)	(−0.07–0.02)
		a *p* = 0.23	a *p* = 0.02	a *p* = 0.94	a *p* = 0.98	a *p* = 0.99	a *p* = 0.55	a *p* = 0.26
		*p* = 0.12	*p* = 0.02	*p* = 0.63	*p* = 0.74	*p* = 0.44	*p* = 0.43	*p* = 0.54
rs1424233	*MAF*	0.02	−0.02	−0.01	−0.03	−0.01	0.004	0.002
		(−0.002–0.03)	(−0.05–0.02)	(−0.02–0.002)	(−0.08–0.02)	(−0.04–0.02)	(−0.02–0.03)	(−0.04–0.05)
		a *p* = 0.07	a *p* = 0.34	a *p* = 0.1	a *p* = 0.28	a *p* = 0.47	a *p* = 0.77	a *p* = 0.92
		*p* = 0.02	*p* = 0.50	*p* = 0.03	*p* = 0.43	*p* = 0.69	*p* = 0.97	*p* = 0.46
rs10508503	*PTER*	0.004	0.03	0.02	0.01	0.005	−0.0005	−0.04
		(−0.03–0.04)	(−0.04–0.09)	(−0.004–0.04)	−0.07–0.1)	(−0.04–0.05)	(−0.05–0.05)	(−0.1–0.05)
		a *p* = 0.79	a *p* = 0.38	a *p* = 0.11	a *p* = 0.77	a *p* = 0.83	a *p* = 0.99	a *p* = 0.39
		*p* = 0.66	*p* = 0.36	*p* = 0.19	*p* = 0.72	*p* = 0.66	*p* = 0.89	*p* = 0.49

Data are presented as effect sizes (95% CI) per allele and *p*-values.

a Effect sizes are adjusted for age, sex and BMI and given as absolute values for total cholesterol and HDL-cholesterol, LDL-cholesterol, and as percentages for triglyceride, leptin, adiponectin and CRP since these traits are logarithmically transformed. LDL-cholesterol is calculated as: ((total cholesterol – HDL-cholesterol – triglyceride/2.2). *p*-values are specified for the additive model and adjusted for age, sex and BMI. *P*-values without ^a^ are only adjusted for age and sex. CRP, C-reactive protein.

**Table 4:** Effects sizes of the obesity risk alleles on measures of glucose homeostasis, with and without adjustments for BMI in the population-based Inter99 cohort.

		Measures of glucose homeostasis			
SNP	Locus	Fasting plasma glucose (mmol/l)	Fasting serum insulin (pmol/l)	HOMA-IR (mmol/lxpmol/l)	Insulinogenic index (pmolxpmol^−1^)
rs7561317	*TMEM18*	0.001	−0.03	−0.03	−0.02
		(−0.004–0.007)	(−0.06-(−0.005))	(−0.05-(−0.002))	(−0.05–0.01)
		a *p* = 0.58	a *p* = 0.02	a *p* = 0.04	a *p* = 0.28
		*p* = 0.06	*p* = 0.65	*p* = 0.42	*p* = 0.95
rs7498665	*SH2B1*	0.0002	0.005	0.006	0.006
		(−0.004–0.004)	(−0.13–0.02)	(−0.01–0.02)	(−0.17–0.03)
		a *p* = 0.94	a *p* = 0.56	a *p* = 0.57	a *p* = 0.62
		*p* = 0.73	*p* = 0.34	*p* = 0.33	*p* = 0.50
rs29941	*KCTD15*	0.002	0.005	0.007	0.003
		(−0.002–0.006)	(−0.01–0.02)	(−0.01–0.03)	(−0.02–0.03)
		a *p* = 0.42	a *p* = 0.62	a *p* = 0.51	a *p* = 0.80
		*p* = 0.20	*p* = 0.20	*p* = 0.15	*p* = 0.61
rs2568958	*NEGR1*	0.003	−0.02	−0.01	−0.02
		(−0.0005–0.007)	(−0.05–0.002)	(−0.03–0.007)	(−0.05-(−0.002))
		a *p* = 0.09	a *p* = 0.08	a *p* = 0.21	a *p* = 0.04
		*p* = 0.06	*p* = 0.27	*p* = 0.52	*p* = 0.07
rs7647305	*ETV5*	0.0008	0.003	0.004	−0.002
		(−0.004–0.006)	(−0.02–0.03)	(−0.02–0.03)	(−0.03–0.03)
		a *p* = 0.74	a *p* = 0.79	a *p* = 0.78	a *p* = 0.89
		*p* = 0.36	*p* = 0.24	*p* = 0.22	*p* = 0.72
rs4923461	*BDNF*	−0.003	−0.01	−0.01	0.01
		(−0.007–0.002)	(−0.03–0.01)	(−0.04–0.01)	(−0.02–0.04)
		a *p* = 0.26	a *p* = 0.41	a *p* = 0.34	a *p* = 0.38
		*p* = 0.84	*p* = 0.50	*p* = 0.52	*p* = 0.13
rs925946	*BDNF*	0.004	−0.01	−0.008	−0.009
		(−0.0002–0.008)	(−0.03–0.008)	(−0.03–0.01)	(−0.03–0.02)
		a *p* = 0.06	a *p* = 0.22	a *p* = 0.47	a *p* = 0.48
		*p* = 0.003	*p* = 0.40	*p* = 0.18	*p* = 0.87
rs10913469	*SEC16B*	0.001	−0.02	−0.02	0.009
		(−0.003–0.006)	(−0.04–0.006)	(−0.04–0.008)	(−0.02–0.04)
		a *p* = 0.60	a *p* = 0.14	a *p* = 0.19	a *p* = 0.52
		*p* = 0.14	*p* = 0.71	*p* = 0.56	*p* = 0.16
rs7138803	*FAIM2*	−0.0005	−0.002	−0.002	0.006
		(−0.004–0.003)	(−0.02–0.02)	(−0.02–0.02)	(−0.02–0.03)
		a *p* = 0.79	a *p* = 0.82	a *p* = 0.82	a *p* = 0.63
		*p* = 0.23	*p* = 0.02	*p* = 0.02	*p* = 0.09
rs10938397	*GNPDA2*	−0.001	0.006	0.004	0.01
		(−0.005–0.003)	(−0.01–0.02)	(−0.02–0.02)	(−0.01–0.04)
		a *p* = 0.51	a *p* = 0.56	a *p* = 0.70	a *p* = 0.28
		*p* = 0.72	*p* = 0.03	*p* = 0.04	*p* = 0.08
rs10838738	*MTCH2*	−0.0002	−0.01	−0.01	−0.007
		(−0.004–0.004)	(−0.03–0.007)	(−0.03–0.008)	(−0.03–0.02)
		a *p* = 0.94	a *p* = 0.21	a *p* = 0.22	a *p* = 0.56
		*p* = 0.82	*p* = 0.18	*p* = 0.19	*p* = 0.45
rs2260000	*BAT2*	−0.003	0.0003	−0.004	−0.001
		(−0.007–0.0009)	(−0.02–0.02)	a *p* = 0.71	(−0.02–0.02)
		a *p* = 0.13	a *p* = 0.97	(−0.02–0.02)	a *p* = 0.92
		*p* = 0.10	*p* = 0.53	*p* = 0.76	*p* = 0.31
rs1805081	*NPC1*	0.0008	−0.01	−0.01	0.01
		(−0.003–0.005)	(−0.03–0.004)	(−0.03–0.005)	(−0.009–0.04)
		a *p* = 0.70	a *p* = 0.13	a *p* = 0.15	a *p* = 0.24
		*p* = 0.45	*p* = 0.45	*p* = 0.52	*p* = 0.24
rs1424233	*MAF*	−0.001	−0.001	−0.002	0.02
		(−0.005–0.003)	(−0.02–0.02)	(−0.02–0.02)	(−0.003–0.04)
		a *p* = 0.56	a *p* = 0.90	a *p* = 0.85	a *p* = 0.09
		*p* = 0.99	*p* = 0.41	*p* = 0.42	*p* = 0.04
rs10508503	*PTER*	−0.002	0.02	0.01	−0.02
		(−0.009–0.004)	(−0.02–0.05)	(−0.02–0.05)	(−0.06–0.02)
		a *p* = 0.50	a *p* = 0.33	a *p* = 0.48	a *p* = 0.30
		*p* = 0.64	*p* = 0.24	*p* = 0.34	*p* = 0.42

Data are presented as effect sizes (95% CI) per allele and *p*-values.

a Effect sizes are adjusted for age, sex and BMI and are given as percentages since the traits are logarithmically transformed, and *p*-values are specified for the additive model and adjusted for age, sex and BMI. *P*-values without ^a^ are only adjusted for age and sex. Homeostasis model assessment of insulin resistance (HOMA-IR) was calculated as ((fasting plasma glucose (mmol/l)×fasting serum insulin (pmol/l))/22.5). Insulinogenic index was calculated as ((serum insulin 30 minuttes post-oral glucose tolerance test (pmol/l) – fasting serum insulin (pmol/l)/plasma glucose 30 minuttes post-oral glucose tolerance test (mmol/l)).

## Discussion

In the second obesity GWAS wave 15 variants in/near 14 loci were identified, and several independent studies have already attempted replication of these findings. Here in our Danish study population we aim to validate these associations, and to elucidate whether some of the variants associate with other clinically relevant phenotypes. Our analyses show the same trends towards increased obesity risk, for all variants except rs10508503 near *PTER*. We report significant associations with overweight and/or obesity for the risk variants in/near *BDNF*, *TMEM18*, *ETV5* and *GNPDA2*. In extended follow-up analyses of anthropometric and metabolic traits in the population-based Inter99 cohort we identified associations with risk alleles in/near *BDNF*, *TMEM18*, *GNPDA2*, *SEC16B* and *FAIM2* when no adjustments for BMI were made. The effect sizes in our Danish study population were generally somewhat higher than for the discovery study (e.g. 0.52 kg/m^2^ vs. 0.26 kg/m^2^ for *TMEM18* rs7561317 and 0.28 kg/m^2^ vs. 0.19 kg/m^2^ for *GNPDA2* rs10938397 [20]), but the pattern of *TMEM18* yielding the highest effect sizes is true both in our study and the discovery studies [19], [20]. *FAIM2* rs7138803 on the other hand show the second highest effect size on BMI in our study, whereas it exerts a more modest effect in the discovery study [19].

As obesity is a strong risk factor of type 2 diabetes we also examined associations with risk of type 2 diabetes, and unexpectedly established a BMI-dependent borderline association with the *BDNF* rs4923461 obesity risk allele and *reduced* risk of type 2 diabetes. An additional association with risk of type 2 diabetes, though increasing the risk as expected, was established for the variant in *SH2B1*. This latter association was nominally independent of BMI adjustments.

Other studies have also confirmed the associations between quantitative measures of obesity, i.e. BMI, weight and waist circumference, and/or the risk of obesity for variants in/near *TMEM18* [23]–[27], SEC16B [27], *NEGR1* [24], [27]–[29], *SH2B1* [26], [28], [30], *MTCH2* [24], [28], [29], *GNPDA2* [25], [28], [30], *FAIM2* [25], [27], [30], *BDNF* [25], [27], [29], and *KCTD15* [29], [30], however, no convincing pattern exist between the verified variants in the performed studies, which could be caused by low power for detecting modest effect sizes, and therefore, as the number of independent replication studies increases, a meta-analyses may be needed to determine true positive associations. The risk of type 2 diabetes has only been addressed in two other studies, and here association between *TMEM18* [28], *GNPDA2* [28], [30], *ETV5*, *FAIM2* and *SH2B1* [30] and a BMI-dependent increased risk of type 2 diabetes have been reported. Hence, we are the first to report a tendency towards an increased risk of type 2 diabetes independent of BMI for *SH2B1*, and rs7498665 could therefore be suggested as a type 2 diabetes variant if this association is replicated in other independent studies. Particularly surprising from our analyses was the finding that the obesity risk allele of *BDNF* (rs4923461) was borderline associated with a reduced risk of type 2 diabetes when adjusted for BMI. However, these divergent associations were not explained by an interaction between the variant and BMI.

Interestingly, both *SH2B1* and *BDNF* are obvious candidate genes for metabolic disorders based on the biological roles of the encoded proteins. That is, the SH2B1 adaptor protein is involved in several signal transduction processes, including the signalling mediated by the binding of insulin and leptin [31]. The association with type 2 diabetes is in line with previous studies showing insulin resistance in knockout mice [32]–[35]. Furthermore, a recent study demonstrated that TgKO mice, that only express SH2B1 in the brain and thus have loss of peripheral SH2B1, have impaired insulin sensitivity independent of body weight [35]. However, none of the examined measures of glucose homeostasis supported a possible reduction in insulin sensitivity in our study.

BDNF, brain-derived neurotrophic factor, is implicated in the regulation of body weight [36]. Yet, our results could suggest more ways for action of the BDNF protein. This is also supported by two studies of db/db mice. One where exogenous BDNF treatment reduces blood glucose concentrations independently of the hypophagic effect [37] and another where subcutaneous BDNF resulted in reduced blood glucose levels in BDNF administered mice, whereas pair-fed control mice displayed unchanged levels, despite that plasma insulin levels were significantly reduced in both groups [38].

Consistent with animal models, plasma BDNF levels have previously been reported to be inversely correlated with fasting plasma glucose among type 2 diabetes patients and to be associated with the severity of insulin resistance [39]. This suggests that BDNF regulates blood glucose homeostasis and insulin sensitivity peripherally. Hence, tissue specific up-regulation of endogenous BDNF levels in peripheral tissues could explain this BMI-dependent protective effect on type 2 diabetes for the reported *BDNF* obesity risk allele (rs4923461).

Contradictory, quantitative trait analyses show that the *other* risk allele near *BDNF* (rs925946) associated with elevated and *not* decreased fasting plasma glucose levels in our study population. Serum leptin and the inflammatory marker CRP were also found to be significantly elevated among rs925946 T-allele carriers in the present study. However, these associations omitted when adjusting for BMI and is therefore most likely mediated through the increased body fat accumulation, rather than being the cause of it.

Different neuronal and peripheral regulatory mechanisms of BDNF could be explained by different isoforms of the protein, and in fact tissue specific alternative splicing has been reported for BDNF in humans [40], [41]. Furthermore, the *BDNFOS* gene, which is a non-protein-coding natural antisense transcript positioned downstream of *BDNF* in reverse orientation, has been suggested to have an important role in tissue specific regulation of *BDNF* expression through the formation of dsRNA duplexes [41]. In fact both gene variants are positioned within the *BDNFOS* locus. Thus, it could be hypothesized that the risk alleles could result in a muscle specific impairment of *BDNFOS* transcription and splicing, which would lead to a reduction in complementary *BDNF* RNAs and consequently increase the level of BDNF. Therefore physiological and experimental studies to illuminate the tissue specific and functional role of *BDNF* and *BDNFOS* will be of great interest.

Of the remaining associated obesity risk loci the potential functional role is less obvious as little is known of the encoded proteins. Furthermore, some of the identified risk variants are next to additional loci, in which the risk variants may influence as well. However, most of the identified loci are highly expressed in the brain, particularly in the hypothalamic region, indicating roles in appetite regulation and energy expenditure [19], [20], [22].

Many analyses were made in this study and some should therefore be regarded as hypothesis-generating with confirmation in independent studies as an important next step, but these analyses in the population-based Inter99 cohort still contribute to the follow-up of the reported findings of the second obesity GWAS wave.

It should moreover be noted that individuals from the Inter99 cohort (Note S1) were included in the follow-up of the top 43 variants in the BMI GWAS performed by Thorleifsson *et al.* However, since these studies were pooled with other ethnicities, we found it necessary to elucidate whether the associations with type 2 diabetes and obesity were present in an ethnically homogeneous study to avoid confounding by population stratification. In view of that, little overlap is observed in associations with type 2 diabetes suggesting that the effect of the BMI risk variants on type 2 diabetes risk is dependent on geographic origin. Moreover, we have in the present study examined the underlying metabolic phenotypes profoundly in order to shed light on the possible metabolic mechanisms causing the reported associations. The Inter99 cohort is in addition used in the replication part of the newest approach in identifying BMI associated variants, the meta-analyses of ∼125,000 individuals and independent replication in the same number, performed by the GIANT (Genomewide Investigation of ANThropometric measures) consortium, revealing 18 new BMI associated loci [42].

In conclusion, of the variants found to associate with obesity and related traits in the second GWAS wave, we were able to report association with obesity and/or measures of adiposity for variants in/near *BDNF*, *TMEM18*, *ETV5*, *GNPDA2*, *SEC16B* and *FAIM2*. Moreover, we found that *SH2B1* rs7498665 strongly associated with type 2 diabetes in a BMI-independent manner. Our analyses also suggested that although rs4923461 in *BDNF* increase the risk of obesity, it conversely protect against type 2 diabetes, which could be through different neuronal and peripheral mechanisms.

## Materials and Methods

### Study populations

The 15 SNPs from the second obesity GWAS wave were genotyped in 18,014 individuals ascertained from four different study groups; 1) the Inter99 cohort, which is a population-based, randomised, non-pharmacological intervention study of middle-aged individuals for the prevention of ischemic heart disease (*n* = 6,514), conducted at the Research Centre for Prevention and Health in Glostrup, Copenhagen (ClinicalTrials.gov ID-no: NCT00289237) [43]; 2) the ADDITION Denmark screening study cohort (Anglo–Danish–Dutch Study of Intensive Treatment in People with Screen-Detected Diabetes in Primary Care) (ClinicalTrials.gov ID-no: NCT00237548) [44], which is a population-based, high-risk screening and intervention study for type 2 diabetes in general practice (*n* = 8,664); 3) a population-based group of unrelated middle-aged individuals (*n* = 680) examined at Steno Diabetes Center; and 4) unrelated type 2 diabetic patients (*n* = 2,158) sampled through the out-patient clinic at Steno Diabetes Center. In BMI stratified case-control analyses individuals from study group 2 with BMI<25 kg/m^2^ were excluded. Hence, in the combined study sample 3,512 were normal weight, 7,458 were overweight, 5,044 were obese, and 340 were morbidly obese. Study groups 1 and 3 underwent a standard 75 g oral glucose tolerance test. Association with type 2 diabetes was evaluated in the combined study sample of which 5,302 were glucose-tolerant and 3,778 were type 2 diabetes patients. Definitions of overweight, obesity, morbid obesity and type 2 diabetes were according to WHO criteria, i.e. 25–29.99 kg/m^2^, 30–39.99 kg/m^2^ and ≥40 kg/m^2^ respectively. In analyses of overweight individuals with BMI<25.0 kg/m^2^ were compared with individuals in the BMI range 25–29.99 kg/m^2^, obesity were analysed comparing BMI<25.0 kg/m^2^ with the 30–39.99 kg/m^2^ range, and finally analyses of morbid obesity were made with individuals with BMI<25.0 kg/m^2^ and individuals with BMI≥40 kg/m^2^.

Of note, a total of 5,586 individuals from study group 1 and 5,450 individuals from study group 2 were included in the GWAS performed by Thorleifsson *et al.* [19] (Note S1).

All study participants were Danes by self report, and informed written consent was obtained from all individuals before participation. The studies were approved by the regional Ethical Committees (The Scientific Ethics Committee of the Capital Region of Denmark for study group 1, 3 and 4 and The Scientific Ethics Committee of the Central region of Denmark for study group 2) and were in accordance with the principles of the Helsinki Declaration. More details of the study groups are given in Table S1.

### Biochemical and anthropometric measures

In all four study groups weight and height were measured in light indoor clothes and without shoes. Waist circumference (cm) was measured in standing position midway between the iliac crest and the lower costal margin. For evaluation of quantitative traits all analysis are performed in the Inter99 cohort as this cohort represents the general middle-aged Danish population and extensive phenotypic characterisations are available in this cohort. Blood samples were drawn after a 12-hour overnight fast. Plasma glucose was analysed by glucose oxidase method (Granutest; Merck, Darmstadt, Germany) and serum insulin (excluding des(31, 32) and intact proinsulin) was measured using the AutoDELFIA insulin kit (Perkin-Elmer, Wallac, Turku, Finland). Serum triglyceride, total cholesterol and HDL-cholesterol were determined using enzymatic colorimetric methods (GPO-PAP and CHOD-PAP; Roche Molecular Biochemicals, Mannheim, Germany). LDL-cholesterol was calculated as: ((total cholesterol – HDL-cholesterol – triglyceride/2.2). Detection antibodies for serum adiponectin (monomeric adiponectin (ADIPOQ)) (R&D Systems; Minneapolis, MN), leptin (R&D Systems; Minneapolis, MN) and C-reactive protein (CRP) (U.S. Biological; Swampscott, MA) were conjugated with Alexa Flour 647 (Invitrogen, Carlsbad, CA) and purified by ultrafiltration with Microcon YM-30 from Millipore (Billerica, MA).

### Genotyping

SNPs for genotyping were selected as described in Note S2. The 10 variants in *TMEM18*, *SH2B1*, *KCTD15*, *NEGR1*, *SEC16B*, *SFRS10*, *BDNF*, *FAIM2* and *BAT2* were genotyped in study group 1 by deCODE genetics using the Centaurus platform [19]. For study group 2–4 these 10 variants were genotyped by KBioscience using the KasPAR® SNP Genotyping method. The remaining 5 variants in *GNPDA2*, *MTCH2*, *NPC1*, *MAF* and *PTER* were genotyped by KBioscience also using the KasPAR® SNP Genotyping method in all four study groups. When adjusting for the multiple tests performed, all SNPs obeyed Hardy Weinberg equilibrium (*p*>0.003). All 15 SNPs passed quality control with an average mismatch rate of 0.17% (max. 0.97%) and an average success rate of 97.8% (min. 96.1%).

### Statistical analyses

Case-control analyses were performed using logistic regression. Type 2 diabetes studies included the full study sample, whereas BMI stratified case-control analyses excluded individuals from study group 2 with BMI<25 kg/m^2^ as controls , since this is a population of high-risk individuals. General linear models were used to test quantitative metabolic traits for differences between genotype groups in 6,039 treatment-naïve individuals from the population-based Inter99 cohort. All analyses were performed assuming an additive genetic model and with adjustments for age and sex. Additionally adjustments for BMI were introduced in case-control studies of type 2 diabetes and in quantitative trait analyses.

Quantitative traits that did not follow a normal distribution were logarithmically transformed.

Statistical power in replication case-control settings was determined using the CaTS power calculator version 0.0.2. The lowest and the highest risk allele frequencies (RAF) of the examined SNPs were 8% and 84%, respectively, and the mean RAF was 50%. Using the population-based Inter99 cohort as reference, the prevalence of overweight, obesity, morbid obesity and type 2 diabetes in the Danish population was estimated to 39%, 17%, 1.3% and 8%, respectively. Estimated statistical power calculations in case-control settings are presented in Table S2.

Statistical power for the quantitative traits was estimated using simulations (*n* = 5,000), where variance across genotypes was drawn from phenotypes simulated to follow normal distribution using empirical variances. The variance for adjustment factors, estimated using residuals of linear models, was also included in the model, assuming independency of genotypes. Linear models were used both for simulating and testing data, assuming additive models and using a significance threshold of 0.05. The estimated statistical power for variants with a RAF of 8%, 84% and 50% respectively, were determined and are presented in Table S3. All analyses were performed in RGui version 2.8.0. Due to the large amount of tests performed in the present study, we used Bonferroni correction for multiple testing. For the primary hypothesis traits this resulted in a significance level of *p*<0.0004, whereas it for follow-up traits resulted in a significance level of *p*<0.0001.

## Supporting Information

Table S1 **Clinical characteristics of the study groups comprising the combined study population.**
(DOCX)

Table S2 **Statistical power calculations in replication case-control settings.**
(DOCX)

Table S3 **Statistical power calculations of quantitative traits.**
(DOCX)

Table S4 **Case-control studies of overweight, obesity and type 2 diabetes in the combined study sample.**
(DOCX)

Table S5 **Anthropometric and metabolic measures in the population-based Inter99 study sample.**
(DOCX)

Note S1 **Individuals included from the Inter99 cohort.**
(DOCX)

Note S2 **SNPs selected for genotyping.**
(DOCX)

## References

[1] Sorensen TI (1989). Genetic and environmental influences on obesity assessed by the adoption method.. Rev Epidemiol Sante Publique.

[2] Stunkard AJ, Harris JR, Pedersen NL, McClearn GE (1990). The body-mass index of twins who have been reared apart.. N Engl J Med.

[3] Maes HH, Neale MC, Eaves LJ (1997). Genetic and environmental factors in relative body weight and human adiposity.. Behav Genet.

[4] Herbert A, Gerry NP, McQueen MB, Heid IM, Pfeufer A (2006). A common genetic variant is associated with adult and childhood obesity.. Science.

[5] Frayling TM, Timpson NJ, Weedon MN, Zeggini E, Freathy RM (2007). A common variant in the FTO gene is associated with body mass index and predisposes to childhood and adult obesity.. Science.

[6] Scuteri A, Sanna S, Chen WM, Uda M, Albai G (2007). Genome-wide association scan shows genetic variants in the FTO gene are associated with obesity-related traits.. PLoS Genet.

[7] Liu YJ, Liu XG, Wang L, Dina C, Yan H (2008). Genome-wide association scans identified CTNNBL1 as a novel gene for obesity.. Hum Mol Genet.

[8] Hinney A, Nguyen TT, Scherag A, Friedel S, Brönner G (2007). Genome wide association (GWA) study for early onset extreme obesity supports the role of fat mass and obesity associated gene (FTO) variants.. PLoS One.

[9] Andreasen CH, Stender-Petersen KL, Mogensen MS, Torekov SS, Wegner L (2008). Low physical activity accentuates the effect of the FTO rs9939609 polymorphism on body fat accumulation.. Diabetes.

[10] Peeters A, Beckers S, Verrijken A, Roevens P, Peeters P (2008). Variants in the FTO gene are associated with common obesity in the Belgian population.. Mol Genet Metab.

[11] Cornes BK, Lind PA, Medland SE, Montgomery GW, Nyholt DR (2009). Replication of the association of common rs9939609 variant of FTO with increased BMI in an Australian adult twin population but no evidence for gene by environment (G×E) interaction.. Int J Obes.

[12] Chambers JC, Elliott P, Zabaneh D, Zhang W, Li Y (2008). Common genetic variation near MC4R is associated with waist circumference and insulin resistance.. Nat Genet.

[13] Loos RJ, Lindgren CM, Li S, Wheeler E, Zhao JH (2008). Common variants near MC4R are associated with fat mass, weight and risk of obesity.. Nat Genet.

[14] Zobel DP, Andreasen CH, Grarup N, Eiberg H, Sørensen TI (2009). Variants near MC4R are associated with obesity and influence obesity-related quantitative traits in a population of middle-aged people: studies of 14,940 Danes.. Diabetes.

[15] Kring SI, Holst C, Toubro S, Astrup A, Hansen T (2010). Common variants near MC4R in relation to body fat, body fat distribution, metabolic traits and energy expenditure.. Int J Obes.

[16] Benzinou M, Creemers JW, Choquet H, Lobbens S, Dina C (2008). Common nonsynonymous variants in PCSK1 confer risk of obesity.. Nat Genet.

[17] Sandholt CH, Sparso T, Grarup N, Albrechtsen A, Almind K (2010). Combined analyses of 20 common obesity susceptibility variants.. Diabetes.

[18] Kilpelainen TO, Bingham SA, Khaw KT, Wareham NJ, Loos RJ (2009). Association of variants in the PCSK1 gene with obesity in the EPIC-Norfolk study.. Hum Mol Genet.

[19] Thorleifsson G, Walters GB, Gudbjartsson DF, Steinthorsdottir V, Sulem P (2009). Genome-wide association yields new sequence variants at seven loci that associate with measures of obesity.. Nat Genet.

[20] Willer CJ, Speliotes EK, Loos RJ, Li S, Lindgren CM (2009). Six new loci associated with body mass index highlight a neuronal influence on body weight regulation.. Nat Genet.

[21] Meyre D, Delplanque J, Chevre JC, Lecoeur C, Lobbens C (2009). Genome-wide association study for early-onset and morbid adult obesity identifies three new risk loci in European populations.. Nat Genet.

[22] Hofker M, Wijmenga C (2009). A supersized list of obesity genes.. Nat Genet.

[23] Almen MS, Jacobsson JA, Shaik JH, Olszewski PK, Cedernaes J (2010). The obesity gene, TMEM18, is of ancient origin, found in majority of neuronal cells in all major brain regions and associated with obesity in severely obese children.. BMC Med Genet.

[24] Haupt A, Thamer C, Heni M, Machicao F, Machann J (2009). Novel Obesity Risk Loci Do Not Determine Distribution of Body Fat Depots: A Whole-body MRI/MRS study.. Obesity.

[25] Hotta K, Nakamura M, Nakamura T, Matsuo T, Nakata Y (2009). Association between obesity and polymorphisms in SEC16B, TMEM18, GNPDA2, BDNF, FAIM2 and MC4R in a Japanese population.. J Hum Genet.

[26] Holzapfel C, Grallert H, Huth C, Wahl S, Fischer B (2010). Genes and lifestyle factors in obesity: results from 12 462 subjects from MONICA/KORA.. Int J Obes.

[27] Scherag A, Dina C, Hinney A, Vatin V, Scherag S (2010). Two New Loci for Body-Weight Regulation Identified in a Joint Analysis of Genome-Wide Association Studies for early-Onset Extreme Obesity in French and German Study Groups.. PLoS Gen.

[28] Renstrom F, Payne F, Nordstrom A, Brito EC, Rolandsson O (2009). Replication and extension of genome-wide association study results for obesity in 4923 adults from northern Sweden.. Hum Mol Genet.

[29] Bauer F, Elbers CC, Adan RA, Loos RJ, Onland-Moret NC (2009). Obesity genes identified in genome-wide association studies are associated with adiposity measures and potentially with nutrient-specific food preference.. Am J Clin Nutr.

[30] Ng MC, Tam CH, So WY, Ho JS, Chan AW (2010). Implication of Genetic Variants Near NEGR1, SEC16B, TMEM18, ETV5/DGKG, GNPDA2, LIN7C/BDNF, MTCH2, BCDIN3D/FAIM2, SH2B1, FTO, MC4R, and KCTD15 with Obesity and Type 2 Diabetes in 7705 Chinese.. J Clin Endocrinol Metab.

[31] Maures TJ, Kurzer JH, Carter-Su C (2007). SH2B1 (SH2-B) and JAK2: a multifunctional adaptor protein and kinase made for each other.. Trends Endocrinol Metab.

[32] Duan C, Yang H, White MF, Rui L (2004). Disruption of the SH2-B gene causes age-dependent insulin resistance and glucose intolerance.. Mol Cell Biol.

[33] Li M, Ren D, Iseki M, Takaki S, Rui L (2006). Differential role of SH2-B and APS in regulating energy and glucose homeostasis.. Endocrinology.

[34] Ren D, Zhou Y, Morris D, Li M, Li Z (2007). Neuronal SH2B1 is essential for controlling energy and glucose homeostasis.. J Clin Invest.

[35] Morris DL, Cho KW, Zhou Y, Rui L (2009). SH2B1 enhances insulin sensitivity by both stimulating the insulin receptor and inhibiting tyrosine dephosphorylation of insulin receptor substrate proteins.. Diabetes.

[36] Xu B, Goulding EH, Zang K, Cepoi D, Cone RD (2003). Brain-derived neurotrophic factor regulates energy balance downstream of melanocortin-4 receptor.. Nat Neurosci.

[37] Nakagawa T, Tsuchida A, Itakura Y, Nonomura T, Ono M (2000). Brain-derived neurotrophic factor regulates glucose metabolism by modulating energy balance in diabetic mice.. Diabetes.

[38] Ono M, Ichihara J, Nonomura T, Itakura Y, Taiji M (1997). Brain-derived neurotrophic factor reduces blood glucose level in obese diabetic mice but not in normal mice.. Biochem Biophys Res Commun.

[39] Krabbe KS, Nielsen AR, Krogh-Madsen R, Plomgaard P, Rasmussen P (2007). Brain-derived neurotrophic factor (BDNF) and type 2 diabetes.. Diabetologia.

[40] Liu QR, Walther D, Drgon T, Polesskaya O, Lesnick TG (2005). Human brain derived neurotrophic factor (BDNF) genes, splicing patterns, and assessments of associations with substance abuse and Parkinson's Disease.. Am J Med Genet B Neuropsychiatr Genet.

[41] Pruunsild P, Kazantseva A, Aid T, Palm K, Timmusk T (2007). Dissecting the human BDNF locus: bidirectional transcription, complex splicing, and multiple promoters.. Genomics.

[42] Speliotes EK, Willer CJ, Berndt SI, Monda KL, Thorleifsson G (2010). Association analyses of 249,796 individuals reveal 18 new loci associated with body mass index.. Nat genet.

[43] Jorgensen T, Borch-Johnsen K, Thomsen TF, Ibsen H, Glumer C (2003). A randomized non-pharmacological intervention study for prevention of ischaemic heart disease: baseline results Inter99.. Eur J Cardiovasc Prev Rehabil.

[44] Lauritzen T, Griffin S, Borch-Johnsen K, Wareham NJ, Wolffenbuttel BH (2000). The ADDITION study: proposed trial of the cost-effectiveness of an intensive multifactorial intervention on morbidity and mortality among people with Type 2 diabetes detected by screening.. Int J Obes Relat Metab Disord.

