# Supplementary material for: Studies of Metabolic Phenotypic Correlates of 15 Obesity Associated Gene Variants
Source: PLoS One. 2011 Sep 2;6(9):e23531. doi: 10.1371/journal.pone.0023531 (PMC3166286; doi:10.1371/journal.pone.0023531)
Supplement: Table S1 — Clinical characteristics of the study groups comprising the combined study population. (DOCX) [file pone.0023531.s001.docx]

**Table S1. Clinical characteristics of the study groups comprising the combined study population.**

|  | **Inter99** | **ADDITION** | **Steno -** | **Steno –** |
| --- | --- | --- | --- | --- |
|  |  | **Denmark Screening cohort** | **glucose-tolerant individuals** | **Type 2 diabetic cases** |
| ***N*** | 6,784 | 8,662 | 521 | 2,111 |
| **Men/women** | 3,070/3,094 | 4,729/3,933 | 244/277 | 1,287/824 |
| **Age** | 46.2 ± 7.9 | 60.0 ± 6.8 | 56.6 ± 10.0 | 62.1 ± 11.3 |
| **(years)** |  |  |  |  |
| **BMI** | 26.3 ± 4.6 | 28.6 ± 4.9 | 25.8 ± 3,7 | 30.1 ± 5.6 |
| **(kg/m^2^)** |  |  |  |  |
| **HbA_1c_** | 5.9 ± 0.6 | 5.9 ± 0.7 | 5.8 ± 0.5 | 8.1 ± 1.6 |
| **(%)** |  |  |  |  |
| **Fasting glucose** | 5.6 ± 1.1 | 5.4 ± 1.2^a^ | 5.1 ± 0.4 | 9.7 ± 3.5 |
| **(mmol/l)** |  |  |  |  |
| **Waist** | 87 ± 13 | 97 ± 14 | 87 ± 11 | 104 ± 15 |
| **(cm)** |  |  |  |  |
| **Serum cholesterol** | 5.5 ± 1.1 | 5.8 ± 1.1 | 6.0 ± 1.1 | 5.7 ±1 .2 |
| **(mmol/l)** |  |  |  |  |
| **Serum HDL-cholesterol** | 1.4 ± 0.4 | 1.6 ± 0.4 | 1.5 ± 0.4 | 1.1 ± 0.3 |
| **(mmol/l)** |  |  |  |  |
| **Serum triglyceride** | 1.3 ± 1.3 | NA | 1.3 ± 0.7 | 2.2 ± 1.9 |
| **(mmol/l)** |  |  |  |  |
| **Systolic BP** | 131 ± 18 | NA | 130 ± 17 | 146 ± 23 |
| **(mmHg)** |  |  |  |  |
| **Diastolic BP** | 83 ± 11 | NA | 77 ± 10 | 83 ± 11 |
| **(mmHg)** |  |  |  |  |

Data are means ± standard deviation. ^a^In the ADDITION study fasting glucose is measured on capillary blood. BP, blood pressure; NA, not available.
