# Supplementary material for: Studies of Metabolic Phenotypic Correlates of 15 Obesity Associated Gene Variants
Source: PLoS One. 2011 Sep 2;6(9):e23531. doi: 10.1371/journal.pone.0023531 (PMC3166286; doi:10.1371/journal.pone.0023531)
Supplement: Table S2 — Statistical power calculations in replication case-control settings. (DOCX) [file pone.0023531.s002.docx]

**Table S2.** **Statistical power calculations in replication case-control settings.**

| **Trait** | **Relative risk (OR)** | **RAF (%)** | **Statistical power (%)** |
| --- | --- | --- | --- |
| Overweight | 1.18 | 8 | 100 |
|  | 1.18 | 84 | 100 |
|  | 1.12 | 50 | 100 |
| Obesity | 1.18 | 8 | 84 |
|  | 1.18 | 84 | 92 |
|  | 1.12 | 50 | 94 |
| Morbid obesity | 1.40 | 8 | 36 |
|  |  | 84 | 43 |
|  |  | 50 | 85 |
| Type 2 diabetes | 1.11 | 8 | 30 |
|  |  | 84 | 47 |
|  |  | 50 | 84 |

Statistical power in replication case-control settings of overweight, obesity, morbid obesity and type 2 diabetes using the population-based Inter99 cohort as reference of prevalence of overweight (39%), obesity (17%), morbid obesity (1.3%), and type 2 diabetes (8%). Analyses are carried out assuming an additive model and using a significance threshold of 0.05.Relative risk is presented as odds ratios (OR). RAF; risk-allele frequency.
