# Supplementary material for: Studies of Metabolic Phenotypic Correlates of 15 Obesity Associated Gene Variants
Source: PLoS One. 2011 Sep 2;6(9):e23531. doi: 10.1371/journal.pone.0023531 (PMC3166286; doi:10.1371/journal.pone.0023531)
Supplement: Table S3 — Statistical power calculations of quantitative traits. (DOCX) [file pone.0023531.s003.docx]

**Table S3. Statistical power calculations of quantitative traits.**

| **Trait** | **Effect** | **RAF (%)** | **Statistical power (%)** |
| --- | --- | --- | --- |
| BMI | 0.3 | 8 | 51 |
|  |  | 84 | 76 |
| (kg½/m^2^) |  | 50 | 96 |
| Fasting plasma glucose | 0.05 | 8 | 44 |
|  |  | 84 | 70 |
| (mmol/l) |  | 50 | 92 |
| Fasting serum insulin | 1.5 | 8 | 35 |
|  |  | 84 | 60 |
| (pmol/l) |  | 50 | 85 |
| Insulinogenic index | 1.0 | 8 | 33 |
|  |  | 84 | 54 |
| (pmol x mmol^-1^) |  | 50 | 80 |
| HOMA-IR | 0.5 | 8 | 46 |
|  |  | 84 | 72 |
| (mmol/l x pmol/l) |  | 50 | 93 |
| Triglycerides | 0.08 | 8 | 44 |
|  |  | 84 | 66 |
| (mmol/l) |  | 50 | 91 |
| Total cholesterol | 0.06 | 8 | 38 |
|  |  | 84 | 62 |
| (mmol/l) |  | 50 | 87 |
| HDL-cholesterol | 0.03 | 8 | 61 |
|  |  | 84 | 87 |
| (mmol/l) |  | 50 | 98 |

Statistical power of quantitative traits estimated using simulations (*n*=5,000), where variance across genotypes was drawn from phenotypes simulated to follow normal distribution using empirical variances. The variance for adjustment factors, estimated using residuals of linear models, was also included in the model, assuming independency of genotypes. Linear models were used both for simulating and testing data, assuming additive models and using a significance threshold of 0.05.RAF; risk-allele frequency
