# Supplementary material for: Studies of Metabolic Phenotypic Correlates of 15 Obesity Associated Gene Variants
Source: PLoS One. 2011 Sep 2;6(9):e23531. doi: 10.1371/journal.pone.0023531 (PMC3166286; doi:10.1371/journal.pone.0023531)
Supplement: Table S4 — Case-control studies of overweight, obesity and type 2 diabetes in the combined study sample. (DOCX) [file pone.0023531.s004.docx]

**Table S4. Case-control studies of overweight, obesity and type 2 diabetes in the combined study sample.**

| ***TMEM18*** | ***n*** | **Genotype distribution** | **RAF** | ***p*_add_** | **OR_add_** |
| --- | --- | --- | --- | --- | --- |
| **rs7561317** | **(men/women)** | ***n* AA/AG/GG** | **(95% CI)** |  | **(95% CI)** |
| **Controls** | 3,345 | 98/981/2,266 | 82.4 |  |  |
|  | (1,337/2,008) |  | (81.5-83.3) |  |  |
| **Overweight cases** | 7,220 | 223/1,979/5,018 | 83.2 | 0.08 | 1.08 |
|  | (4,492/2,728) |  | (82.6-83.8) |  | (0.99-1.17) |
| **Obese cases** | 4,898 | 118/1,248/3,532 | 84.9 | 2.1 x 10^-6^ | 1.25 |
|  | (2,529/2,369) |  | (84.1-85.6) |  | (1.14-1.37) |
| **Morbid obese cases** | 398 | 6/91/301 | 87.1 | 8.3 x 10^-4^ | 1.46 |
|  | (133/265) |  | (84.5-89.3) |  | (1.17-1.82) |
| **Controls** | 4961 | 144/1,371/3,446 | 83.3 |  |  |
|  | (2,310/2,651) |  | (82.5-84) |  |  |
| **T2D** | 3,549 | 88/919/2,542 | 84.6 | 0.02 | 1.13 |
| **patients** | (2,104/1,445) |  | (83.7-85.4) |  | (1.02-1.26) |
| ^a^BMI adjustment: OR_add_ (95% CI)=1.04 (0.92-1.17), *p*_add_=0.52 | | | | | |
| ***SH2B1*** | ***n*** | **Genotype distribution** | **RAF** | ***p*_add_** | **OR_add_** |
| **rs7498665** | **(men/women)** | ***n* AA/AG/GG** | **(95% CI)** |  | **(95% CI)** |
| **Controls** | 3,315 | 1,183/1,541/591 | 41.1 |  |  |
|  | (1,322/1,993) |  | (39.9-42.3) |  |  |
| **Overweight cases** | 7,166 | 2,453/3,478/1,235 | 41.5 | 0.74 | 1.01 |
|  | (4,447/2,719) |  | (40.7-42.3) |  | (0.95-1.08) |
| **Obese cases** | 4,857 | 1,602/2,377/878 | 42.5 | 0.12 | 1.06 |
|  | (2,516/2,341) |  | (41.6-43.5) |  | (0.99-1.13) |
| **Morbid obese cases** | 394 | 111/209/74 | 45.3 | 0.05 | 1.17 |
|  | (131/263) |  | (41.8-48.9) |  | (1.00-1.36) |
| **Controls** | 4,916 | 1,791/2,287/838 | 40.3 |  |  |
|  | (2,283/2,633) |  | (39.3-41.3) |  |  |
| **T2D** | 3,528 | 1,133/1,738/657 | 43.3 | 3.0 x 10^-5^ | 1.18 |
| **patients** | (2,096/1,432) |  | (42.1-44.4) |  | (1.09-1.28) |
| ^a^BMI adjustment: OR_add_ (95% CI)=1.16 (1.07-1.27), *p*_add_=7.8 x 10^-4^ | | | | | |
| ***KCTD15*** | ***n*** | **Genotype distribution** | **RAF** | ***p*_add_** | **OR_add_** |
| **rs29941** | **(men/women)** | ***n* AA/AG/GG** | **(95% CI)** |  | **(95% CI)** |
| **Controls** | 3,288 | 367/1,410/1,511 | 67.4 |  |  |
|  | (1,323/1,965) |  | (66.2-68.5) |  |  |
| **Overweight cases** | 7,134 | 773/3,081/3,280 | 67.6 | 0.93 | 1.00 |
|  | (4,444/2,690) |  | (66.8-68.3) |  | (0.94-1.07) |
| **Obese cases** | 4,860 | 526/2,071/2,263 | 67.9 | 0.60 | 1.02 |
|  | (2,514/2,346) |  | (66.9-68.8) |  | (0.95-1.10) |
| **Morbid obese cases** | 397 | 38/163/196 | 69.9 | 0.30 | 1.09 |
|  | (133/264) |  | (66.6-73.1) |  | (0.93-1.28) |
| **Controls** | 4,880 | 519/2,085/2,276 | 68.0 |  |  |
|  | (2,284/2,596) |  | (67.1-68.9) |  |  |
| **T2D** | 3,522 | 368/1,539/1,615 | 67.7 | 0.64 | 0.98 |
| **patients** | (2,098/1,424) |  | (66.6-68.8) |  | (0.90-1.07) |
| ^a^BMI adjustment: OR_add_ (95% CI)=0.99 (0.90-1.09), *p*_add_=0.79 | | | | | |
| ***NEGR1*** | ***n*** | **Genotype distribution** | **RAF** | ***p*_add_** | **OR_add_** |
| **rs2568958** | **(men/women)** | ***n* GG/GA/AA** | **(95% CI)** |  | **(95% CI)** |
| **Controls** | 3,33 | 560/1,608/1,168 | 59.1 |  |  |
|  | (1,332/2,004) |  | (57.9-60.3) |  |  |
| **Overweight cases** | 7,204 | 1,227/3,418/2,559 | 59.2 | 0.75 | 1.01 |
|  | (4,486/2,718) |  | (58.4-60.0) |  | (0.95-1.08) |
| **Obese cases** | 4,901 | 803/2,298/1,800 | 60.2 | 0.28 | 1.04 |
|  | (2,530/2,371) |  | (59.2-61.1) |  | (0.97-1.11) |
| **Morbid obese cases** | 396 | 64/178/154 | 61.4 | 0.28 | 1.09 |
|  | (132/264) |  | (57.9-64.8) |  | (0.93-1.27) |
| **Controls** | 4,946 | 858/2,338/1,750 | 59.0 |  |  |
|  | (2,301/2,645) |  | 58.0-60.0 |  |  |
| **T2D** | 3,554 | 584/1,683/1,287 | 59.9 | 0.05 | 1.08 |
| **patients** | (2,113/1,441) |  | (58.7-61.0) |  | (1.00-1.17) |
| ^a^BMI adjustment: OR_add_ (95% CI)=1.06 (0.97-1.16), *p*_add_=0.20 | | | | | |
| ***ETV5*** | ***n*** | **Genotype distribution** | **RAF** | ***p*_add_** | **OR_add_** |
| **rs7647305** | **(men/women)** | ***n* TT/TC/CC** | **(95% CI)** |  | **(95% CI)** |
| **Controls** | 3,274 | 136/1,043/2,095 | 79.9 |  |  |
|  | (1,306/1,968) |  | (78.9-80.9) |  |  |
| **Overweight cases** | 7,090 | 254/2,125/4,711 | 81.4 | 0.02 | 1.10 |
|  | (4,418/2,672) |  | (80.8-82.1) |  | (1.02-1.19) |
| **Obese cases** | 4,825 | 157/1,368/3,300 | 82.6 | 1.8 x 10^-4^ | 1.18 |
|  | (2,491/2,334) |  | (81.8-83.3) |  | (1.08-1.29) |
| **Morbid obese cases** | 394 | 9/100/285 | 85.0 | 0.001 | 1.41 |
|  | (134/260) |  | (82.3-87.4) |  | (1.15-1.74) |
| **Controls** | 4,852 | 191/1,517/3,144 | 80.4 |  |  |
|  | (2,261/2,591) |  | (79.6-81.2) |  |  |
| **T2D** | 3,492 | 128/1,009/2,355 | 81.9 | 0.02 | 1.12 |
| **patients** | (2,074/1,418) |  | (81.0-82.8) |  | (1.02-1.24) |
| ^a^BMI adjustment: OR_add_ (95% CI)=1.04 (0.93-1.16), *p*_add_=0.50 | | | | | |
| ***BDNF*** | ***n*** | **Genotype distribution** | **RAF** | ***p*_add_** | **OR_add_** |
| **rs4923461** | **(men/women)** | ***n* GG/GA/AA** | **(95% CI)** |  | **(95% CI)** |
| **Controls** | 3,339 | 175/1,237/1,927 | 76.2 |  |  |
|  | (1,335/2,004) |  | (75.2-77.3) |  |  |
| **Overweight cases** | 7,125 | 348/2,440/4,337 | 78.0 | 2.5 x 10^-4^ | 1.15 |
|  | (4,440/2,685) |  | (77.3-78.7) |  | (1.07-1.24) |
| **Obese cases** | 4,839 | 232/1,649/2,958 | 78.2 | 0.002 | 1.14 |
|  | (2,498/2,341) |  | (77.3-79.0) |  | (1.05-1.23) |
| **Morbid obese cases** | 395 | 25/122/248 | 78.2 | 0.21 | 1.12 |
|  | (132/263) |  | (75.2-81.1) |  | (0.94-1.35) |
| **Controls** | 4,957 | 241/1,724/2,992 | 77.8 |  |  |
|  | (2,308/2,649) |  | 76.9-78.6 |  |  |
| **T2D** | 3,508 | 195/1,228/2,085 | 77.0 | 0.4 | 0.96 |
| **patients** | (2,084/1,424) |  | (76.0-77.9) |  | (0.87-1.05) |
| ^a^BMI adjustment: OR_add_ (95% CI)=0.87 (0.78-0.96), *p*_add_=0.008 | | | | | |
| ***BDNF*** | ***n*** | **Genotype distribution** | **RAF** | ***p*_add_** | **OR_add_** |
| **rs925946** | **(men/women)** | ***n* GG/GT/TT** | **(95% CI)** |  | **(95% CI)** |
| **Controls** | 3,307 | 1,674/1,372/261 | 28.6 |  |  |
|  | (1,316/1,991) |  | (27.5-29.7) |  |  |
| **Overweight cases** | 7,178 | 3,268/3,142/768 | 32.6 | 1.4 x 10^-7^ | 1.20 |
|  | (4,472/2,706) |  | (31.8-33.4) |  | (1.12-1.29) |
| **Obese cases** | 4,869 | 2,282/2,114/473 | 31.4 | 3.9 x 10^-4^ | 1.15 |
|  | (2,515/2,354) |  | (30.5-32.4) |  | (1.06-1.24) |
| **Morbid obese cases** | 394 | 180/177/37 | 31.9 | 0.08 | 1.16 |
|  | (132/262) |  | (28.6-35.2) |  | (0.98-1.37) |
| **Controls** | 4,906 | 2,340/2,120/446 | 30.7 |  |  |
|  | (2,292/2,614) |  | (29.8-31.6) |  |  |
| **T2D** | 3,541 | 1,676/1,502/363 | 31.4 | 0.5 | 0.97 |
| **patients** | (2,102/1,439) |  | (30.4-32.5) |  | (0.89-1.06) |
| ^a^BMI adjustment: OR_add_ (95% CI)=0.91 (0.83-1.00), *p*_add_=0.06 | | | | | |
| ***SEC16B*** | ***n*** | **Genotype distribution** | **RAF** | ***p*_add_** | **OR_add_** |
| **rs10913469** | **(men/women)** | ***n* TT/TC/CC** | **(95% CI)** |  | **(95% CI)** |
| **Controls** | 3,359 | 2,151/1,071/137 | 20.0 |  |  |
|  | (1,341/2,018) |  | (19.1-21.0) |  |  |
| **Overweight cases** | 7,290 | 4,615/2,361/314 | 20.5 | 0.32 | 1.04 |
|  | (4,552/2,738) |  | (19.8-21.2) |  | (0.96-1.12) |
| **Obese cases** | 4,953 | 3,013/1,700/240 | 22.0 | 0.01 | 1.11 |
|  | (2,557/2,396) |  | (21.2-22.8) |  | (1.03-1.21) |
| **Morbid obese cases** | 400 | 243/139/18 | 21.9 | 0.25 | 1.11 |
|  | (135/265) |  | (19.1-24.9) |  | (0.93-1.34) |
| **Controls** | 4,972 | 3,096/1,642/234 | 21.2 |  |  |
|  | (2,321/2,651) |  | (20.4-22.0) |  |  |
| **T2D** | 3,619 | 2,277/1,189/153 | 20.7 | 0.47 | 0.97 |
| **patients** | (2,150/1,469) |  | (19.7-21.6) |  | (0.88-1.06) |
| ^a^BMI adjustment: OR_add_ (95% CI)=0.90 (0.81-1.00), *p*_add_=0.05 | | | | | |
| ***FAIM2*** | ***n*** | **Genotype distribution** | **RAF** | ***p*_add_** | **OR_add_** |
| **rs7138803** | **(men/women)** | ***n* GG/GA/AA** | **(95% CI)** |  | **(95% CI)** |
| **Controls** | 3,338 | 1,230/1,571/537 | 39.6 |  |  |
|  | (1,337/2,001) |  | (38.4-40.8) |  |  |
| **Overweight cases** | 7,221 | 2,578/3,413/1,230 | 40.7 | 0.33 | 1.03 |
|  | (4,495/2,726) |  | (39.9-41.5) |  | (0.97-1.10) |
| **Obese cases** | 4,893 | 1,671/2,379/843 | 41.5 | 0.01 | 1.09 |
|  | (2,523/2,370) |  | (40.6-42.5) |  | (1.02-1.17) |
| **Morbid obese cases** | 398 | 116/211/71 | 44.3 | 0.03 | 1.19 |
|  | (133/265) |  | (40.9-47.9) |  | (1.02-1.38) |
| **Controls** | 4,933 | 1,822/2,291/820 | 39.8 |  |  |
|  | (2,299/2,634) |  | (38.9-40.8) |  |  |
| **T2D** | 3,592 | 1,237/1,749/606 | 41.2 | 0.06 | 1.08 |
| **patients** | (2,134/1,458) |  | (40.1-42.4) |  | (1.00-1.17) |
| ^a^BMI adjustment: OR_add_ (95% CI)=1.06 (0.97-1.15), *p*_add_=0.23 | | | | | |
| ***GNPDA2*** | ***N*** | **Genotype distribution** | **RAF** | ***p*_add_** | **OR_add_** |
| **rs10938397** | **(men/women)** | ***n* TT/TC/CC** | **(95% CI)** |  | **(95% CI)** |
| **Controls** | 3,291 | 1,204/1,582/505 | 39.4 |  |  |
|  | (1,308/1,983) |  | (38.2-40.6) |  |  |
| **Overweight cases** | 7,131 | 2,502/3,416/1,213 | 41.0 | 0.01 | 1.09 |
|  | (4,443/2,688) |  | (40.2-41.8) |  | (1.02-1.16) |
| **Obese cases** | 4,835 | 1,591/2,377/867 | 42.5 | 1.1 x 10^-4^ | 1.15 |
|  | (2,492/2,343) |  | (41.5-43.5) |  | (1.07-1.23) |
| **Morbid obese cases** | 396 | 129/187/80 | 43.8 | 0.02 | 1.20 |
|  | (132/264) |  | (40.3-47.3) |  | (1.03-1.4) |
| **Controls** | 4,873 | 1,715/2,329/829 | 40.9 |  |  |
|  | (2,259/2,614) |  | (39.9-41.9) |  |  |
| **T2D** | 3,461 | 1,134/1,741/586 | 42.1 | 0.19 | 1.06 |
| **patients** | (2,053/1,408) |  | (40.9-43.3) |  | (0.97-1.14) |
| ^a^BMI adjustment: OR_add_ (95% CI)=0.99 (0.90-1.08), *p*_add_=0.79 | | | | | |
| ***MTCH2*** | ***n*** | **Genotype distribution** | **RAF** | ***p*_add_** | **OR_add_** |
| **rs10838738** | **(men/women)** | ***n* AA/AG/GG** | **(95% CI)** |  | **(95% CI)** |
| **Controls** | 3,295 | 1,398/1,485/412 | 35.0 |  |  |
|  | (1,318/1,977) |  | (33.9-36.2) |  |  |
| **Overweight cases** | 7,131 | 2,990/3,263/878 | 35.2 | 0.91 | 1.00 |
|  | (4,448/2,683) |  | (34.4-36.0) |  | (0.94-1.07) |
| **Obese cases** | 4,850 | 2,081/2,127/642 | 35.2 | 0.86 | 1.01 |
|  | (2,497/2,353) |  | (34.2-36.1) |  | (0.94-1.08) |
| **Morbid obese cases** | 390 | 171/167/52 | 34.7 | 0.94 | 0.99 |
|  | (130/260) |  | (31.4-38.2) |  | (0.85-1.17) |
| **Controls** | 4,881 | 2,062/2,216/603 | 35.1 |  |  |
|  | (2,271/2,610) |  | (34.1-36.0) |  |  |
| **T2D** | 3,502 | 1,451/1,577/474 | 36.1 | 0.34 | 1.04 |
| **patients** | (2,077/1,425) |  | (34.9-37.2) |  | (0.96-1.13) |
| ^a^BMI adjustment: OR_add_ (95% CI)=1.03 (0.94-1.13), *p*_add_=0.49 | | | | | |
| ***BAT2*** | ***n*** | **Genotype distribution** | **RAF** | ***p*_add_** | **OR_add_** |
| **rs2260000** | **(men/women)** | ***n* CC/CT/TT** | **(95% CI)** |  | **(95% CI)** |
| **Controls** | 2,869 | 436/1,263/1,170 | 62.8 |  |  |
|  | (1,163/1,706) |  | (61.5-64.0) |  |  |
| **Overweight cases** | 6,691 | 848/3,092/2,751 | 64.2 | 0.10 | 1.06 |
|  | (4,162/2,529) |  | (63.4-65.0) |  | (0.99-1.14) |
| **Obese cases** | 4,656 | 641/2,120/1,895 | 63.5 | 0.81 | 1.01 |
|  | (2,404/2,252) |  | (62.5-64.4) |  | (0.94-1.09) |
| **Morbid obese cases** | 379 | 54/161/164 | 64.5 | 0.51 | 1.05 |
|  | (124/255) |  | (61.0-67.9) |  | (0.90-1.24) |
| **Controls** | 4,123 | 572/1,834/1,717 | 63.9 |  |  |
|  | (1,913/2,210) |  | (62.8-64.9) |  |  |
| **T2D** | 3,467 | 486/1,612/1,369 | 62.7 | 0.08 | 0.93 |
| **patients** | (2,056/1,411) |  | (61.6-63.9) |  | (0.85-1.01) |
| ^a^BMI adjustment: OR_add_ (95% CI)=0.91 (0.83-1.00), *p*_add_=0.05 | | | | | |
| ***NPC1*** | ***N*** | **Genotype distribution** | **RAF** | ***p*_add_** | **OR_add_** |
| **rs1805081** | **(men/women)** | ***n* GG/GA/AA** | **(95% CI)** |  | **(95% CI)** |
| **Controls** | 3,352 | 625/1,675/1,052 | 56.4 |  |  |
|  | (1,338/2,014) |  | (55.2-57.6) |  |  |
| **Overweight cases** | 7,196 | 1,299/3,507/2,390 | 57.6 | 0.07 | 1.06 |
|  | (4,481/2,715) |  | (56.8-58.4) |  | (1.00-1.13) |
| **Obese cases** | 4,894 | 853/2,341/1,700 | 58.7 | 0.005 | 1.10 |
|  | (2,526/2,368) |  | (57.7-59.6) |  | (1.03-1.18) |
| **Morbid obese cases** | 397 | 67/185/145 | 59.8 | 0.02 | 1.21 |
|  | (134/263) |  | (56.3-63.3) |  | (1.03-1.41) |
| **Controls** | 4,937 | 917/2,421/1,599 | 56.9 |  |  |
|  | (2,298/2,639) |  | (55.9-57.9) |  |  |
| **T2D** | 3,579 | 621/1,756/1,202 | 58.1 | 0.03 | 1.09 |
| **patients** | (2,124/1,455) |  | (57.0-59.3) |  | (1.01-1.18) |
| ^a^BMI adjustment: OR_add_ (95% CI)=1.07 (0.98-1.17), *p*_add_=0.13 | | | | | |
| ***MAF*** | ***N*** | **Genotype distribution** | **RAF** | ***p*_add_** | **OR_add_** |
| **rs1424233** | **(men/women)** | ***n* AA/AG/GG** | **(95% CI)** |  | **(95% CI)** |
| **Controls** | 3,317 | 958/1,654/705 | 46.2 |  |  |
|  | (1,325/1,992) |  | (45.0-47.4) |  |  |
| **Overweight cases** | 7,176 | 2,013/3,531/1,632 | 47.3 | 0.11 | 1.05 |
|  | (4,473/2,703) |  | (46.5-48.2) |  | (0.99-1.12) |
| **Obese cases** | 4,891 | 1,341/2,457/1,093 | 47.5 | 0.08 | 1.06 |
|  | (2,530/2,361) |  | (46.5-48.5) |  | (0.99-1.14) |
| **Morbid obese cases** | 391 | 106/189/96 | 48.7 | 0.36 | 1.07 |
|  | (132/259) |  | (45.2-52.3) |  | (0.92-1.25) |
| **Controls** | 4,912 | 1,379/2,436/1,097 | 47.1 |  |  |
|  | (2,292/2,620) |  | (46.1-48.1) |  |  |
| **T2D** | 3,549 | 998/1,790/761 | 46.7 | 0.40 | 0.97 |
| **patients** | (2,111/1,438) |  | (45.5-47.8) |  | (0.89-1.05) |
| ^a^BMI adjustment: OR_add_ (95% CI)=0.94 (0.86-1.02), *p*_add_=0.14 | | | | | |
| ***PTER*** | ***N*** | **Genotype distribution** | **RAF** | ***p*_add_** | **OR_add_** |
| **rs10508503** | **(men/women)** | ***n* CC/CT/TT** | **(95% CI)** |  | **(95% CI)** |
| **Controls** | 3,324 | 2,779/517/28 | 8.6 |  |  |
|  | (1,328/1,996) |  | (8.0-9.3) |  |  |
| **Overweight cases** | 7,237 | 6,094/1,089/54 | 8.3 | 0.29 | 0.94 |
|  | (4,514/2,723) |  | (7.8-8.7) |  | (0.84-1.05) |
| **Obese cases** | 4,918 | 4,129/756/33 | 8.4 | 0.84 | 0.99 |
|  | (2,540/2,378) |  | (7.8-8.9) |  | (0.87-1.12) |
| **Morbid obese cases** | 394 | 326/65/3 | 9.0 | 0.61 | 1.07 |
|  | (133/261) |  | (7.1-11.2) |  | (0.82-1.40) |
| **Controls** | 4,939 | 4,121/778/40 | 8.7 |  |  |
|  | (2,300/2,639) |  | (8.1-9.3) |  |  |
| **T2D** | 3,582 | 2,995/559/28 | 8.6 | 0.33 | 0.93 |
| **patients** | (2,129/1,453) |  | (7.9-9.3) |  | (0.81-1.07) |
| ^a^BMI adjustment: OR_add_ (95% CI)=0.91 (0.78-1.07), *p*_add_=0.26 | | | | | |

Data are presented as distribution of individuals according to genotype, risk-allele frequency (RAF) in % (95% CI), and OR (95% CI) and *p*-values. OR and *p*-values are specified for the additive model and adjusted for age and sex. ^a^Association with type 2 diabetes is additionally adjusted for BMI.
