# Supplementary material for: Studies of Metabolic Phenotypic Correlates of 15 Obesity Associated Gene Variants
Source: PLoS One. 2011 Sep 2;6(9):e23531. doi: 10.1371/journal.pone.0023531 (PMC3166286; doi:10.1371/journal.pone.0023531)
Supplement: Table S5 — Anthropometric and metabolic measures in the population-based Inter99 study sample. (DOCX) [file pone.0023531.s005.docx]

**Table S5.** **Anthropometric and metabolic measures in the population-based Inter99 study sample.**

| ***TMEM18*** | **AA** | **AG** | **GG** | ***p*_add_** | ^a^***p*_add_** |
| --- | --- | --- | --- | --- | --- |
| **rs7561317** |  |  |  |  |  |
| N | 160 | 1,641 | 4,083 |  |  |
| (men/women) | (72/88) | (839/802) | (2,024/2,059) |  |  |
| Age (years) | 47 ± 8 | 46 ± 8 | 46 ± 8 |  |  |
| **Obesity-related measures** | | | | | |
| BMI (kg/m^2^) | 25.5 ± 3.9 | 25.9 ± 4.25 | 26.4 ± 4.65 | 2.9 x 10^-6^ | - |
| Weight (kg) | 75.1 ± 14.7 | 77.3 ± 15.2 | 78.5 ± 16.3 | 3.1 x 10^-5^ | - |
| Height (cm) | 171.2 ± 8.9 | 172.6 ± 9.2 | 172.3 ± 9.2 | 0.65 | - |
| Waist (cm) | 84.5 ± 12.0 | 86.0 ± 12.7 | 86.8 ± 13.4 | 1.8 x 10^-4^ | - |
| Waist-Hip ratio | 0.85 ± 0.08 | 0.86 ± 0.08 | 0.86 ± 0.09 | 0.039 | - |
| **Fasting serum adipokines and CRP** | | | | | |
| Leptin (ng/ml) | 5.4 (2.7;11.5) | 5.3 (2.5;10.8) | 5.8 (2.7;12.1) | 0.01 | 0.87 |
| Adiponectin (ng/ml) | 7,892 (4,620;13,030) | 6,864 (4,142;12,410) | 7,055 (4,146;11,790) | 0.41 | 0.99 |
| CRP (ng/ml) | 1,000 (366;1,892) | 803 (350;1,853) | 864 (385;2,080) | 0.02 | 0.37 |
| **Fasting serum lipids** | | | | | |
| Triglyceride (mmol/l) | 1.33 ± 0.98 | 1.34 ± 1.75 | 1.34 ± 1.18 | 0.23 | 0.67 |
| Total cholesterol (mmol/l) | 5.64 ± 1.00 | 5.53 ± 1.03 | 5.53 ± 1.08 | 1 | 0.51 |
| HDL-cholesterol (mmol/l) | 1.44 ± 0.43 | 1.45 ± 0.41 | 1.43 ± 0.40 | 0.16 | 0.87 |
| LDL-cholesterol (mmol/l) | 3.58 ± 1.02 | 3.53 ± 0.97 | 3.53 ± 0.97 | 0.58 | 0.21 |
| **Glucose homeostasis** | | | | | |
| Fasting plasma glucose (mmol/l) | 5.45 ± 0.56 | 5.53 ± 0.68 | 5.54 ± 0.82 | 0.06 | 0.58 |
| Fasting serum insulin (pmol/l) | 35 (25;49) | 34 (24;51) | 34 (24;51) | 0.65 | 0.02 |
| HOMA-IR (mmol/lxpmol/l) | 8.6 (5.8;11.8) | 8.3 (5.6;12.7) | 8.3 (5.7;13.0) | 0.42 | 0.04 |
| Insulinogenic index (pmolxpmol^-1^) | 31.58 ± 18.94 | 28.47 ± 19.32 | 29.67 ± 19.83 | 0.95 | 0.28 |
| ***SH2B1*** | **AA** | **AG** | **GG** | ***p*_add_** | ^a^***p*_add_** |
| **rs7498665** |  |  |  |  |  |
| N | 2,083 | 2,711 | 1,024 |  |  |
| (men/women) | (1,021/1,062) | (1,371/1,340) | (499/525) |  |  |
| Age (years) | 46 ± 8 | 46 ± 8 | 46 ± 8 |  |  |
| **Obesity-related measures** | | | | | |
| BMI (kg/m^2^) | 26.1 ± 4.5 | 26.3 ± 4.6 | 26.2 ± 4.5 | 0.35 | - |
| Weight (kg) | 77.6 ± 15.7 | 78.5 ± 16.2 | 78.0 ± 15.8 | 0.31 | - |
| Height (cm) | 172.2 ± 9.2 | 172.5 ± 9.2 | 172.2 ± 9.2 | 0.67 | - |
| Waist (cm) | 86.1 ± 12.8 | 86.7 ± 13.5 | 86.4 ± 13.0 | 0.28 | - |
| Waist-Hip ratio | 0.85 ± 0.08 | 0.86 ± 0.09 | 0.86 ± 0.09 | 0.28 | - |
| **Fasting serum adipokines and CRP** | | | | | |
| Leptin (ng/ml) | 5.8 (2.6;12.1) | 5.5 (2.6;11.9) | 5.5 (2.7;11.5) | 0.70 | 0.45 |
| Adiponectin (ng/ml) | 7,292 (4,142;12,770) | 6,976 (4,227;11,820) | 6,821 (4,073;11,770) | 0.09 | 0.09 |
| CRP (ng/ml) | 890 (393;2,106) | 817 (378;2,000) | 821 (344;1,895) | 0.15 | 0.12 |
| **Fasting serum lipids** | | | | | |
| Triglyceride (mmol/l) | 1.1 (0.8;1.6) | 1.1 (0.8;1.6) | 1.0 (0.7;1.5) | 0.32 | 0.17 |
| Total cholesterol (mmol/l) | 5.56 ± 1.08 | 5.5 ± 1.03 | 5.55 ± 1.11 | 0.54 | 0.45 |
| HDL-cholesterol (mmol/l) | 1.44 ± 0.41 | 1.43 ± 0.40 | 1.45 ± 0.41 | 0.39 | 0.22 |
| LDL-cholesterol (mmol/l) | 3.55 ± 0.98 | 3.5 ± 0.96 | 3.55 ± 0.99 | 0.59 | 0.67 |
| **Glucose homeostasis** | | | | | |
| Fasting plasma glucose (mmol/l) | 5.52 ± 0.80 | 5.55 ± 0.75 | 5.52 ± 0.82 | 0.73 | 0.94 |
| Fasting serum insulin (pmol/l) | 34 (23;41) | 35 (24;43) | 34 (24;42) | 0.34 | 0.56 |
| HOMA-IR (mmol/lxpmol/l) | 8.1 (5.6;12.6) | 8.5 (5.8;13.2) | 8.2 (5.5;12.5) | 0.33 | 0.57 |
| Insulinogenic index (pmolxpmol^-1^) | 28.96 ± 18.27 | 29.67 ± 21.13 | 29.39 ± 18.45 | 0.50 | 0.62 |
| ***KCTD15*** | **AA** | **AG** | **GG** | ***p*_add_** | ^a^***p*_add_** |
| **rs29941** |  |  |  |  |  |
| N | 631 | 2,436 | 2,720 |  |  |
| (men/women) | (307/324) | (1,240/1,196) | (1,347/1,373) |  |  |
| Age (years) | 46 ± 8 | 46 ± 8 | 46 ± 8 |  |  |
| **Obesity-related measures** | | | | | |
| BMI (kg/m^2^) | 26.0 ± 4.4 | 26.2 ± 4.5 | 26.3 ± 4.6 | 0.08 | - |
| Weight (kg) | 77.4 ± 15.5 | 78.0 ± 15.7 | 78.4 ± 16.2 | 0.06 | - |
| Height (cm) | 172.3 ± 9.4 | 172.4 ± 9.3 | 172.3 ± 9.2 | 0.71 | - |
| Waist (cm) | 86.0 ± 12.9 | 86.5 ± 13.0 | 86.7 ± 13.4 | 0.19 | - |
| Waist-Hip ratio | 0.85 ± 0.09 | 0.86 ± 0.08 | 0.86 ± 0.09 | 0.49 | - |
| **Fasting serum adipokines and CRP** | | | | | |
| Leptin (ng/ml) | 5.7 (2.4;11.9) | 5.5 (2.6;11.6) | 5.7 (2.7;11.9) | 0.37 | 0.94 |
| Adiponectin (ng/ml) | 6,943 (4,192;12,410) | 7,095 (4,211;11,840) | 6,998 (4,107;12,080) | 0.82 | 0.97 |
| CRP (ng/ml) | 842 (354;1,793) | 857 (372;2,127) | 827 (383;1,893) | 0.79 | 0.31 |
| **Fasting serum lipids** | | | | | |
| Triglyceride (mmol/l) | 1.0 (0.7;1.5) | 1.1 (0.8;1.6) | 1.1 (0.8;1.6) | 0.15 | 0.37 |
| Total cholesterol (mmol/l) | 5.47 ± 1.09 | 5.52 ± 1.05 | 5.55 ± 1.07 | 0.13 | 0.20 |
| HDL-cholesterol (mmol/l) | 1.44 ± 0.40 | 1.43 ± 0.40 | 1.43 ± 0.41 | 0.58 | 0.99 |
| LDL-cholesterol (mmol/l) | 3.51 ± 0.97 | 3.51 ± 0.95 | 3.55 ± 0.99 | 0.34 | 0.50 |
| **Glucose homeostasis** | | | | | |
| Fasting plasma glucose (mmol/l) | 5.51 ± 0.62 | 5.52 ± 0.8 | 5.55 ± 0.8 | 0.20 | 0.42 |
| Fasting serum insulin (pmol/l) | 34 (24;49) | 35 (24;50) | 35 (24;52) | 0.2 | 0.62 |
| HOMA-IR (mmol/lxpmol/l) | 8.0 (5.7;12.8) | 8.3 (5.5;12.7) | 8.5 (5.7;13.2) | 0.15 | 0.51 |
| Insulinogenic index (pmolxpmol^-1^) | 28.38 ± 17.92 | 29.49 ± 19.38 | 29.54 ± 20.14 | 0.61 | 0.80 |
| ***NEGR1*** | **GG** | **GA** | **AA** | ***p*_add_** | ^a^***p*_add_** |
| **rs2568958** |  |  |  |  |  |
| N | 1,003 | 2,758 | 2,094 |  |  |
| (men/women) | (501/502) | (1,389/1,369) | (1,025/1,069) |  |  |
| Age (years) | 46 ± 8 | 46 ± 8 | 46 ± 8 |  |  |
| **Obesity-related measures** | | | | | |
| BMI (kg/m^2^) | 26.2 ± 4.6 | 26.2 ± 4.5 | 26.3 ± 4.6 | 0.46 | - |
| Weight (kg) | 77.0 ± 15.8 | 78.1 ± 15.7 | 78.2 ± 16.5 | 0.44 | - |
| Height (cm) | 172.2 ± 9.2 | 172.5 ± 9.1 | 172.1 ± 9.3 | 0.88 | - |
| Waist (cm) | 86.4 ± 13.2 | 86.5 ± 13.1 | 86.5 ± 13.3 | 0.42 | - |
| Waist-Hip ratio | 0.85 ± 0.09 | 0.86 ± 0.09 | 0.86 ± 0.09 | 0.09 | - |
| **Fasting serum adipokines and CRP** | | | | | |
| Leptin (ng/ml) | 5.5 (2.8;11.1) | 5.5 (2.6;11.8) | 5.6 (2.7;12.1) | 0.24 | 0.40 |
| Adiponectin (ng/ml) | 6,634 (4,061;11,290) | 7,195 (4,239;12,140) | 7,095 (4,126;12,310) | 0.86 | 0.75 |
| CRP (ng/ml) | 903 (389;2,024) | 828 (367;1,917) | 855 (385;2,106) | 0.84 | 0.96 |
| **Fasting serum lipids** | | | | | |
| Triglyceride (mmol/l) | 1.1 (0.8;1.6) | 1.0 (0.8;1.5) | 1.1 (0.8;1.6) | 0.29 | 0.16 |
| Total cholesterol (mmol/l) | 5.58 ± 1.07 | 5.53 ± 1.08 | 5.51 ± 1.06 | 0.16 | 0.13 |
| HDL-cholesterol (mmol/l) | 1.41 ± 0.39 | 1.44 ± 0.40 | 1.44 ± 0.41 | 0.12 | 0.06 |
| LDL-cholesterol (mmol/l) | 3.62 ± 0.99 | 3.54 ± 0.97 | 3.48 ± 0.97 | 0.009 | 0.01 |
| **Glucose homeostasis** | | | | | |
| Fasting plasma glucose (mmol/l) | 5.54 ± 0.81 | 5.51 ± 0.77 | 5.56 ± 0.78 | 0.06 | 0.09 |
| Fasting serum insulin (pmol/l) | 35 (25;53) | 34 (24;51) | 35 (24;51) | 0.27 | 0.08 |
| HOMA-IR (mmol/lxpmol/l) | 8.5 (5.9;13.2) | 8.2 (5.6;12.9) | 8.4 (5.7;12.9) | 0.52 | 0.21 |
| Insulinogenic index (pmolxpmol^-1^) | 30.05 ± 18.80 | 29.34 ± 19.83 | 29.23 ± 20.00 | 0.07 | 0.04 |
| ***ETV5*** | **TT** | **TC** | **CC** | ***p*_add_** | ^a^***p*_add_** |
| **rs7647305** |  |  |  |  |  |
| N | 233 | 1,779 | 3,737 |  |  |
| (men/women) | (125/108) | (876/903) | (1,867/,1870) |  |  |
| Age (years) | 47 ± 8 | 46 ± 8 | 46 ± 8 |  |  |
| **Obesity-related measures** | | | | | |
| BMI (kg/m^2^) | 26.1 ± 4.4 | 26.0 ± 4.4 | 26.3 ± 4.6 | 0.04 | - |
| Weight (kg) | 77.7 ± 16.1 | 77.6 ± 15.5 | 78.4 ± 16.2 | 0.04 | - |
| Height (cm) | 172.3 ± 9.1 | 172.3 ± 9.1 | 172.4 ± 9.2 | 0.57 | - |
| Waist (cm) | 86.3 ± 13.8 | 85.9 ± 12.8 | 86.8 ± 13.3 | 0.008 | - |
| Waist-Hip ratio | 0.86 ± 0.09 | 0.85 ± 0.09 | 0.86 ± 0.09 | 0.02 | - |
| **Fasting serum adipokines and CRP** | | | | | |
| Leptin (ng/ml) | 5.5 (2.3;10.8) | 5.5 (2.6;11.7) | 5.7 (2.7;11.9) | 0.05 | 0.49 |
| Adiponectin (ng/ml) | 6,539 (4,234;10,720) | 6,987 (4,199;12,280) | 7,095 (4,169;12,020) | 0.76 | 0.46 |
| CRP (ng/ml) | 885 (347;2,124) | 843 (356;1,967) | 843 (384;1,985) | 0.82 | 0.52 |
| **Fasting serum lipids** | | | | | |
| Triglyceride (mmol/l) | 1.1 (0.8;1.6) | 1.0 (0.8;1.5) | 1.1 (0.8;1.6) | 0.14 | 0.40 |
| Total cholesterol (mmol/l) | 5.51 ± 1.15 | 5.5 ± 1.07 | 5.54 ± 1.06 | 0.12 | 0.20 |
| HDL-cholesterol (mmol/l) | 1.4 ± 0.38 | 1.44 ± 0.40 | 1.43 ± 0.40 | 0.81 | 0.35 |
| LDL-cholesterol (mmol/l) | 3.55 ± 1.01 | 3.56 ± 1.02 | 3.52 ± 0.95 | 0.42 | 0.31 |
| **Glucose homeostasis** | | | | | |
| Fasting plasma glucose (mmol/l) | 5.66 ± 1.10 | 5.50 ± 0.74 | 5.55 ± 0.78 | 0.36 | 0.74 |
| Fasting serum insulin (pmol/l) | 35 (23;51) | 34 (23;50) | 35 (24;51) | 0.24 | 0.79 |
| HOMA-IR (mmol/lxpmol/l) | 8.5 (5.5;13.2) | 8.1 (5.5;12.6) | 8.4 (5.7;13.2) | 0.22 | 0.78 |
| Insulinogenic index (pmolxpmol^-1^) | 27.81 ± 20.72 | 29.62 ± 20.03 | 29.37 ± 19.53 | 0.72 | 0.89 |
| ***BDNF*** | **GG** | **GA** | **AA** | ***p*_add_** | ^a^***p*_add_** |
| **rs4923461** |  |  |  |  |  |
| N | 268 | 2,025 | 3,580 |  |  |
| (men/women) | (130/138) | (980/1,045) | (1,819/1,761) |  |  |
| Age (years) | 46 ± 8 | 46 ± 8 | 46 ± 8 |  |  |
| **Obesity-related measures** | | | | | |
| BMI (kg/m^2^) | 25.9 ± 4.6 | 26.0 ± 4.5 | 26.4 ± 4.5 | 0.004 | - |
| Weight (kg) | 77.3 ± 15.9 | 77.2 ± 16.1 | 78.7 ± 15.9 | 0.005 | - |
| Height (cm) | 172.5 ± 9.5 | 172.0 ± 9.2 | 172.5 ± 9.2 | 0.81 | - |
| Waist (cm) | 85.9 ± 12.9 | 85.8 ± 13.2 | 86.9 ± 13.2 | 0.02 | - |
| Waist-Hip ratio | 0.86 ± 0.09 | 0.85 ± 0.08 | 0.86 ± 0.09 | 0.63 | - |
| **Fasting serum adipokines** | | | | | |
| Leptin (ng/ml) | 5.8 (2.7;13.2) | 5.5 (2.7;11.2) | 5.6 (2.6;12.2) | 0.22 | 0.65 |
| Adiponectin (ng/ml) | 6,553 (4,221;11,440) | 7,022 (4,205;12,410) | 7,036 (4,108;11,840) | 0.92 | 0.73 |
| CRP (ng/ml) | 798 (428;2,343) | 789 (366;1,840) | 872 (381;2,105) | 0.13 | 0.48 |
| **Fasting serum lipids** | | | | | |
| Triglyceride (mmol/l) | 1.1 (0.8;1.6) | 1.0 (0.8;1.5) | 1.1 (0.8;1.6) | 0.92 | 0.34 |
| Total cholesterol (mmol/l) | 5.56 ± 1.21 | 5.53 ± 1.09 | 5.53 ± 1.04 | 0.88 | 0.57 |
| HDL-cholesterol (mmol/l) | 1.41 ± 0.41 | 1.45 ± 0.40 | 1.43 ± 0.40 | 0.68 | 0.54 |
| LDL-cholesterol (mmol/l) | 3.58 ± 1.09 | 3.54 ± 0.99 | 3.52 ± 0.96 | 0.39 | 0.28 |
| **Glucose homeostasis** | | | | | |
| Fasting plasma glucose (mmol/l) | 5.54 ± 0.77 | 5.53 ± 0.79 | 5.54 ± 0.77 | 0.84 | 0.26 |
| Fasting serum insulin (pmol/l) | 34 (24;52) | 34 (25;50) | 35 (24;52) | 0.50 | 0.41 |
| HOMA-IR (mmol/lxpmol/l) | 8.1 (5.6;12.6) | 8.3 (5.7;12.6) | 8.4 (5.7;13.2) | 0.52 | 0.34 |
| Insulinogenic index (pmolxpmol^-1^) | 27.87 ± 15.98 | 29.05 ± 19.35 | 29.76 ± 20.08 | 0.13 | 0.38 |
| ***BDNF*** | **GG** | **GT** | **TT** | ***p*_add_** | ^a^***p*_add_** |
| **rs925946** |  |  |  |  |  |
| N | 2,753 | 2,495 | 552 |  |  |
| (men/women) | (1,323/1,430) | (1,271/1,224) | (301/251) |  |  |
| Age (years) | 46 ± 8 | 46 ± 8 | 47 ± 8 |  |  |
| **Obesity-related measures** | | | | | |
| BMI (kg/m^2^) | 26.0 ± 4.5 | 26.4 ± 4.5 | 26.9 ± 4.6 | 8.2 x 10^-5^ | - |
| Weight (kg) | 77.1 ± 15.9 | 78.8 ± 15.9 | 79.9 ± 16.1 | 2.4 x 10^-4^ | - |
| Height (cm) | 172.0 ± 9.2 | 172.6 ± 9.2 | 172.5 ± 8.9 | 0.9 | - |
| Waist (cm) | 85.8 ± 13.0 | 86.8 ± 13.3 | 88.5 ± 13.4 | 8.7 x 10^-4^ | - |
| Waist-Hip ratio | 0.85 ± 0.09 | 0.86 ± 0.09 | 0.87 ± 0.09 | 0.23 | - |
| **Fasting serum adipokines** | | | | | |
| Leptin (ng/ml) | 5.5 (2.6;11.5) | 5.6 (2.6;11.6) | 5.9 (2.9;13.4) | 8.3 x 10^-4^ | 0.24 |
| Adiponectin (ng/ml) | 6,975 (4,176;12,190) | 7,002 (4,089;11,650) | 7,059 (4,475;12,450) | 0.52 | 0.18 |
| CRP (ng/ml) | 836 (368;1,948) | 810 (369;1,923) | 1,088 (456;2,487) | 0.006 | 0.13 |
| **Fasting serum lipids** | | | | | |
| Triglyceride (mmol/l) | 1.1 (0.8;1.5) | 1.0 (0.8;1.5) | 1.1 (0.8;1.6) | 0.55 | 0.04 |
| Total cholesterol (mmol/l) | 5.53 ± 1.08 | 5.53 ± 1.07 | 5.58 ± 1.02 | 0.93 | 0.53 |
| HDL-cholesterol (mmol/l) | 1.44 ± 0.40 | 1.44 ± 0.41 | 1.41 ± 0.38 | 0.93 | 0.21 |
| LDL-cholesterol (mmol/l) | 3.52 ± 0.97 | 3.55 ± 0.99 | 3.55 ± 0.93 | 0.90 | 0.60 |
| **Glucose homeostasis** | | | | | |
| Fasting plasma glucose (mmol/l) | 5.5 ± 0.72 | 5.54 ± 0.8 | 5.65 ± 0.96 | 0.003 | 0.06 |
| Fasting serum insulin (pmol/l) | 34 (24;51) | 34 (24;51) | 35 (25;54) | 0.40 | 0.22 |
| HOMA-IR (mmol/lxpmol/l) | 8.2 (5.7;12.6) | 8.4 (5.6;12.9) | 8.6 (5.9;14.2) | 0.18 | 0.47 |
| Insulinogenic index (pmolxpmol^-1^) | 29.68 ± 20.61 | 29.23 ± 18.84 | 29.50 ± 19.25 | 0.87 | 0.48 |
| ***SEC16B*** | **TT** | **TC** | **CC** | ***p*_add_** | ^a^***p*_add_** |
| **rs10913469** |  |  |  |  |  |
| N | 3,671 | 1,937 | 275 |  |  |
| (men/women) | (1,831/1,840) | (975/962) | (134/141) |  |  |
| Age (years) | 46 ± 8 | 46 ± 8 | 46 ± 8 |  |  |
| **Obesity-related measures** | | | | | |
| BMI (kg/m^2^) | 26.1 ± 4.5 | 26.4 ± 4.6 | 26.6 ± 4.8 | 9.2 x 10^-4^ | - |
| Weight (kg) | 77.7 ± 15.8 | 78.9 ± 16.4 | 78.6 ± 15.0 | 0.005 | - |
| Height (cm) | 172.3 ± 9.2 | 172.4 ± 9.2 | 171.9 ± 9.4 | 0.76 | - |
| Waist (cm) | 86.1 ± 13.1 | 87.2 ± 13.4 | 87.1 ± 12.6 | 0.001 | - |
| Waist-Hip ratio | 0.85 ± 0.09 | 0.86 ± 0.09 | 0.85 ± 0.08 | 0.05 | - |
| **Fasting serum adipokines** | | | | | |
| Leptin (ng/ml) | 5.5 (2.6;11.6) | 5.6 (2.7;12.0) | 5.4 (2.9;11.6) | 0.52 | 0.11 |
| Adiponectin (ng/ml) | 6,996 (4,109;11,990) | 6,978 (4,278;11,900) | 7,454 (4,139;12,650) | 0.75 | 0.36 |
| CRP (ng/ml) | 827 (360;1,914) | 862 (390;2,171) | 905 (436;1,655) | 0.09 | 0.70 |
| **Fasting serum lipids** | | | | | |
| Triglyceride (mmol/l) | 1.0 (0.8;1.5) | 1.1 (0.8;1.6) | 1.1 (0.8;1.5) | 0.07 | 0.49 |
| Total cholesterol (mmol/l) | 5.51 ± 1.06 | 5.57 ± 1.08 | 5.50 ± 1.14 | 0.21 | 0.42 |
| HDL-cholesterol (mmol/l) | 1.44 ± 0.40 | 1.44 ± 0.41 | 1.41 ± 0.39 | 0.52 | 0.61 |
| LDL-cholesterol (mmol/l) | 3.52 ± 0.97 | 3.57 ± 0.99 | 3.41 ± 0.88 | 0.99 | 0.62 |
| **Glucose homeostasis** | | | | | |
| Fasting plasma glucose (mmol/l) | 5.52 ± 0.77 | 5.56 ± 0.82 | 5.51 ± 0.61 | 0.14 | 0.6 |
| Fasting serum insulin (pmol/l) | 35 (24;51) | 34 (24;52) | 35 (23;51) | 0.71 | 0.14 |
| HOMA-IR (mmol/lxpmol/l) | 8.4 (5.7;12.9) | 8.2 (5.7;13.2) | 8.4 (5.6;12.6) | 0.56 | 0.19 |
| Insulinogenic index (pmolxpmol^-1^) | 29.11 ± 19.22 | 29.77 ± 19.96 | 30.61 ± 22.78 | 0.16 | 0.52 |
| ***FAIM2*** | **GG** | **GA** | **AA** | ***p*_add_** | ^a^***p*_add_** |
| **rs7138803** |  |  |  |  |  |
| N | 2,130 | 2,714 | 985 |  |  |
| (men/women) | (1,091/1,039) | (1,337/1,377) | (479/506) |  |  |
| Age (years) | 46 ± 8 | 46 ± 8 | 46 ± 8 |  |  |
| **Obesity-related measures** | | | | | |
| BMI (kg/m^2^) | 25.9 ± 4.2 | 26.2 ± 4.6 | 26.8 ± 4.8 | 8.2 x10^-7^ | - |
| Weight (kg) | 77.2 ± 15.3 | 78.1 ± 16.3 | 79.9 ± 16.4 | 7.6 x10^-8^ | - |
| Height (cm) | 172.3 ± 9.1 | 172.4 ± 9.4 | 172.4 ± 9.0 | 0.05 | - |
| Waist (cm) | 85.9 ± 12.9 | 86.4 ± 13.2 | 87.9 ± 13.6 | 4.9 x10^-7^ | - |
| Waist-Hip ratio | 0.86 ± 0.09 | 0.85 ± 0.09 | 0.86 ± 0.09 | 0.005 | - |
| **Fasting serum adipokines** | | | | | |
| Leptin (ng/ml) | 5.3 (2.5;10.7) | 5.6 (2.6;12.1) | 6.1 (2.9;13.1) | 0.001 | 0.65 |
| Adiponectin (ng/ml) | 7,109 (4,248;11,980) | 6,976 (4,127;12,070) | 6,788 (4,022;11,350) | 0.08 | 0.37 |
| CRP (ng/ml) | 846 (383;1,964) | 847 (360;2,071) | 831 (399;1,920) | 0.66 | 0.18 |
| **Fasting serum lipids** | | | | | |
| Triglyceride (mmol/l) | 1.1 (0.8;1.5) | 1.1 (0.8;1.5) | 1.1 (0.8;1.6) | 0.10 | 0.99 |
| Total cholesterol (mmol/l) | 5.53 ± 1.03 | 5.53 ± 1.09 | 5.53 ± 1.07 | 0.91 | 0.56 |
| HDL-cholesterol (mmol/l) | 1.44 ± 0.41 | 1.44 ± 0.40 | 1.41 ± 0.38 | 0.003 | 0.13 |
| LDL-cholesterol (mmol/l) | 3.49 ± 0.92 | 3.57 ± 1.02 | 3.5 ± 0.96 | 0.61 | 0.71 |
| **Glucose homeostasis** | | | | | |
| Fasting plasma glucose (mmol/l) | 5.53 ± 0.75 | 5.52 ± 0.75 | 5.57 ± 0.92 | 0.23 | 0.79 |
| Fasting serum insulin (pmol/l) | 34 (23;50) | 34 (24;51) | 35 (25;52) | 0.02 | 0.82 |
| HOMA-IR (mmol/lxpmol/l) | 8.2 (5.6;12.6) | 8.3 (5.6;13.0) | 8.6 (5.9;13.2) | 0.02 | 0.82 |
| Insulinogenic index (pmolxpmol^-1^) | 29.22 ± 21.41 | 29.27 ± 18.38 | 30.08 ± 19.42 | 0.09 | 0.63 |
| ***GNPDA2*** | **AA** | **AG** | **GG** | ***p*_add_** | ^a^***p*_add_** |
| **rs10938397** |  |  |  |  |  |
| N | 2,026 | 2,751 | 982 |  |  |
| (men/women) | (987/1,039) | (1,391/1,360) | (483/499) |  |  |
| Age (years) | 46 ± 8 | 46 ± 8 | 46 ± 8 |  |  |
| **Obesity-related measures** | | | | | |
| BMI (kg/m^2^) | 26.0 ± 4.5 | 26.2 ± 4.5 | 26.6 ± 4.6 | 0.001 | - |
| Weight (kg) | 77.3 ± 15.7 | 78.4 ± 16.1 | 78.8 ± 16.2 | 0.006 | - |
| Height (cm) | 172.1 ± 9.0 | 172.6 ± 9.3 | 171.9 ± 9.3 | 0.40 | - |
| Waist (cm) | 85.8 ± 13.1 | 86.7 ± 13.2 | 87.0 ± 13.4 | 0.004 | - |
| Waist-Hip ratio | 0.85 ± 0.09 | 0.86 ± 0.09 | 0.86 ± 0.09 | 0.10 | - |
| **Fasting serum adipokines** | | | | | |
| Leptin (ng/ml) | 5.5 (2.6;11.9) | 5.6 (2.6;11.5) | 5.5 (2.7;12.6) | 0.07 | 0.71 |
| Adiponectin (ng/ml) | 7,039 (4,250;12,030) | 7,027 (4,047;11,970) | 6,933 (4,163;12,070) | 0.79 | 0.37 |
| CRP (ng/ml) | 832 (360;1,871) | 854 (387;2,084) | 861 (375;2,110) | 0.06 | 0.48 |
| **Fasting serum lipids** | | | | | |
| Triglyceride (mmol/l) | 1.1 (0.8;1.5) | 1.1 (0.8;1.6) | 1.0 (0.8;1.5) | 0.98 | 0.24 |
| Total cholesterol (mmol/l) | 5.51 ± 1.06 | 5.52 ± 1.07 | 5.58 ± 1.08 | 0.07 | 0.17 |
| HDL-cholesterol (mmol/l) | 1.45 ± 0.40 | 1.42 ± 0.40 | 1.45 ± 0.40 | 0.96 | 0.22 |
| LDL-cholesterol (mmol/l) | 3.51 ± 0.97 | 3.53 ± 0.97 | 3.57 ± 0.98 | 0.20 | 0.35 |
| **Glucose homeostasis** | | | | | |
| Fasting plasma glucose (mmol/l) | 5.53 ± 0.81 | 5.54 ± 0.80 | 5.53 ± 0.80 | 0.72 | 0.51 |
| Fasting serum insulin (pmol/l) | 34 (23;49) | 34 (24;52) | 35 (24;52) | 0.03 | 0.56 |
| HOMA-IR (mmol/lxpmol/l) | 8.2 (5.5;12.4) | 8.3 (5.7;13.2) | 8.4 (5.8;12.9) | 0.04 | 0.70 |
| Insulinogenic index (pmolxpmol^-1^) | 28.65 ± 18.59 | 29.63 ± 19.87 | 29.60 ± 20.63 | 0.08 | 0.28 |
| ***MTCH2*** | **AA** | **AG** | **GG** | ***p*_add_** | ^a^***p*_add_** |
| **rs10838738** |  |  |  |  |  |
| N | 2,464 | 2,597 | 709 |  |  |
| (men/women) | (1,208/1,256) | (1,321/1,276) | (347/362) |  |  |
| Age (years) | 46 ± 8 | 46 ± 8 | 46 ± 8 |  |  |
| **Obesity-related measures** | | | | | |
| BMI (kg/m^2^) | 26.2 ± 4.6 | 26.2 ± 4.5 | 26.1 ± 4.3 | 0.66 | - |
| Weight (kg) | 78.0 ± 16.2 | 78.4 ± 15.9 | 77.5 ± 15.8 | 0.66 | - |
| Height (cm) | 172.2 ± 9.2 | 172.5 ± 9.1 | 172.2 ± 9.6 | 0.83 | - |
| Waist (cm) | 86.4 ± 13.3 | 86.6 ± 13.1 | 86.2 ± 13.4 | 0.97 | - |
| Waist-Hip ratio | 0.86 ± 0.09 | 0.86 ± 0.09 | 0.86 ± 0.09 | 0.74 | - |
| **Fasting serum adipokines** | | | | | |
| Leptin (ng/ml) | 5.8 (2.6;12.2) | 5.3 (2.6;11.2) | 5.7 (2.8;13.1) | 0.48 | 0.14 |
| Adiponectin (ng/ml) | 7,019 (4,096;12,050) | 6,939 (4,176;11,780) | 7,348 (4,241;12,640) | 0.40 | 0.47 |
| CRP (ng/ml) | 874 (381;1,973) | 830 (360;2,035) | 837 (414;2,011) | 0.83 | 0.98 |
| **Fasting serum lipids** | | | | | |
| Triglyceride (mmol/l) | 1.1 (0.8;1.5) | 1.1 (0.8;1.6) | 1.0 (0.8;1.5) | 0.71 | 0.83 |
| Total cholesterol (mmol/l) | 5.54 ± 1.08 | 5.53 ± 1.07 | 5.47 ± 1.06 | 0.33 | 0.36 |
| HDL-cholesterol (mmol/l) | 1.45 ± 0.41 | 1.42 ± 0.40 | 1.41 ± 0.38 | 0.006 | 0.002 |
| LDL-cholesterol (mmol/l) | 3.53 ± 1.0 | 3.54 ± 0.96 | 3.49 ± 0.92 | 0.59 | 0.54 |
| **Glucose homeostasis** | | | | | |
| Fasting plasma glucose (mmol/l) | 5.54 ± 0.83 | 5.53 ± 0.74 | 5.55 ± 0.92 | 0.82 | 0.94 |
| Fasting serum insulin (pmol/l) | 35 (24;51) | 34 (23;51) | 34 (24;52) | 0.18 | 0.21 |
| HOMA-IR (mmol/lxpmol/l) | 8.5 (5.7;12.9) | 8.1 (5.5;12.7) | 8.2 (5.7;13.3) | 0.19 | 0.22 |
| Insulinogenic index (pmolxpmol^-1^) | 29.75 ± 20.35 | 28.90 ± 19.25 | 29.20 ± 18.08 | 0.45 | 0.56 |
| ***BAT2*** | **CC** | **CT** | **TT** | ***p*_add_** | ^a^***p*_add_** |
| **rs2260000** |  |  |  |  |  |
| N | 809 | 2,695 | 2,383 |  |  |
| (men/women) | (382/427) | (1,363/1,332) | (1,186/1,197) |  |  |
| Age (years) | 46 ± 8 | 46 ± 8 | 46 ± 8 |  |  |
| **Obesity-related measures** | | | | | |
| BMI (kg/m^2^) | 26.1 ± 4.7 | 26.2 ± 4.5 | 26.3 ± 4.4 | 0.39 | - |
| Weight (kg) | 77.2 ± 16.3 | 78.0 ± 15.8 | 78.4 ± 16.0 | 0.03 | - |
| Height (cm) | 171.8 ± 9.0 | 172.2 ± 9.2 | 172.7 ± 9.2 | 0.0006 | - |
| Waist (cm) | 85.9 ± 13.6 | 86.6 ± 13.0 | 86.7 ± 13.4 | 0.18 | - |
| Waist-Hip ratio | 0.86 ± 0.09 | 0.86 ± 0.09 | 0.86 ± 0.09 | 0.74 | - |
| **Fasting serum adipokines** | | | | | |
| Leptin (ng/ml) | 5.4 (2.8;12.7) | 5.5 (2.6;11.8) | 5.7 (2.6;11.6) | 0.84 | 0.70 |
| Adiponectin (ng/ml) | 7,250 (4,147;12,680) | 6,971 (4,149;11,790) | 6,979 (4,210;11,860) | 0.88 | 0.98 |
| CRP (ng/ml) | 868 (394;2,003) | 826 (367;1,932) | 864 (375;2,042) | 0.48 | 0.40 |
| **Fasting serum lipids** | | | | | |
| Triglyceride (mmol/l) | 1.0 (0.8;1.5) | 1.0 (0.8;1.5) | 1.1 (0.8;1.5) | 0.05 | 0.08 |
| Total cholesterol (mmol/l) | 5.53 ± 1.09 | 5.54 ± 1.06 | 5.51 ± 1.07 | 0.63 | 0.71 |
| HDL-cholesterol (mmol/l) | 1.48 ± 0.43 | 1.44 ± 0.39 | 1.44 ± 0.39 | 0.03 | 0.04 |
| LDL-cholesterol (mmol/l) | 3.45 ± 0.93 | 3.55 ± 0.95 | 3.53 ± 1.01 | 0.31 | 0.39 |
| **Glucose homeostasis** | | | | | |
| Fasting plasma glucose (mmol/l) | 5.56 ± 0.73 | 5.57 ± 0.90 | 5.52 ± 0.68 | 0.09 | 0.05 |
| Fasting serum insulin (pmol/l) | 35 (23;53) | 34 (24;51) | 35 (24;52) | 0.72 | 0.95 |
| HOMA-IR (mmol/lxpmol/l) | 8.4 (5.4;13.4) | 8.4 (5.7;12.9) | 8.4 (5.7;13.0) | 0.96 | 0.58 |
| Insulinogenic index (pmolxpmol^-1^) | 29.90 ± 23.10 | 29.45 ± 19.98 | 28.82 ± 18.40 | 0.92 | 0.95 |
| ***NPC1*** | **GG** | **GA** | **AA** | ***p*_add_** | ^a^***p*_add_** |
| **rs1805081** |  |  |  |  |  |
| N | 1,077 | 2,873 | 1,888 |  |  |
| (men/women) | (524/553) | (1,395/1,478) | (990/898) |  |  |
| Age (years) | 46 ± 8 | 46 ± 8 | 46 ± 8 |  |  |
| **Obesity-related measures** | | | | | |
| BMI (kg/m^2^) | 26.2 ± 4.4 | 26.1 ± 4.5 | 26.3 ± 4.6 | 0.18 | - |
| Weight (kg) | 77.9 ± 15.8 | 77.6 ± 15.7 | 78.7 ± 16.4 | 0.48 | - |
| Height (cm) | 172.3 ± 9.1 | 172.2 ± 9.1 | 172.6 ± 9.4 | 0.17 | - |
| Waist (cm) | 86.1 ± 13.1 | 86.2 ± 13.2 | 86.9 ± 13.3 | 0.25 | - |
| Waist-Hip ratio | 0.85 ± 0.09 | 0.85 ± 0.09 | 0.86 ± 0.08 | 0.08 | - |
| **Fasting serum adipokines** | | | | | |
| Leptin (ng/ml) | 5.8 (2.8;11.7) | 5.4 (2.6;11.6) | 5.6 (2.6;11.8) | 0.44 | 0.99 |
| Adiponectin (ng/ml) | 7,186 (4,349;12,430) | 6,980 (4,140;11,850) | 6,892 (4,065;11,840) | 0.43 | 0.55 |
| CRP (ng/ml) | 876 (392;1,957) | 843 (368;2,050) | 822 (368;1,921) | 0.54 | 0.26 |
| **Fasting serum lipids** | | | | | |
| Triglyceride (mmol/l) | 1.1 (0.8;1.5) | 1.1 (0.8;1.5) | 1.1 (0.8;1.6) | 0.12 | 0.23 |
| Total cholesterol (mmol/l) | 5.48 ± 1.05 | 5.53 ± 1.07 | 5.55 ± 1.07 | 0.02 | 0.02 |
| HDL-cholesterol (mmol/l) | 1.44 ± 0.41 | 1.45 ± 0.41 | 1.42 ± 0.39 | 0.63 | 0.94 |
| LDL-cholesterol (mmol/l) | 3.51 ± 0.98 | 3.53 ± 0.99 | 3.53 ± 0.94 | 0.74 | 0.98 |
| **Glucose homeostasis** | | | | | |
| Fasting plasma glucose (mmol/l) | 5.53 ± 0.79 | 5.54 ± 0.83 | 5.54 ± 0.76 | 0.45 | 0.70 |
| Fasting serum insulin (pmol/l) | 35 (24;51) | 34 (24;51) | 34 (24;51) | 0.45 | 0.13 |
| HOMA-IR (mmol/lxpmol/l) | 8.5 (5.8;13.1) | 8.2 (5.6;12.7) | 8.3 (5.7;13.0) | 0.52 | 0.15 |
| Insulinogenic index (pmolxpmol^-1^) | 28.79 ± 19.41 | 29.28 ± 19.35 | 29.67 ± 20.02 | 0.24 | 0.24 |
| ***MAF*** | **AA** | **AG** | **GG** | ***p*_add_** | ^a^***p*_add_** |
| **rs1424233** |  |  |  |  |  |
| N | 1,612 | 2,894 | 1,301 |  |  |
| (men/women) | (810/802) | (1,457/1,437) | (638/663) |  |  |
| Age (years) | 46 ± 8 | 46 ± 8 | 46 ± 8 |  |  |
| **Obesity-related measures** | | | | | |
| BMI (kg/m^2^) | 26.1 ± 4.4 | 26.2 ± 4.6 | 26.4 ± 4.6 | 0.07 | - |
| Weight (kg) | 77.8 ± 15.6 | 78.1 ± 15.9 | 78.6 ± 16.2 | 0.09 | - |
| Height (cm) | 172.4 ± 9.2 | 172.3 ± 9.1 | 172.3 ± 9.4 | 0.98 | - |
| Waist (cm) | 86.4 ± 12.9 | 86.6 ± 13.3 | 86.5 ± 13.2 | 0.44 | - |
| Waist-Hip ratio | 0.86 ± 0.09 | 0.86 ± 0.09 | 0.85 ± 0.09 | 0.72 | - |
| **Fasting serum adipokines** | | | | | |
| Leptin (ng/ml) | 5.5 (2.5;12.2) | 5.5 (2.7;11.2) | 5.5 (2.7;12.3) | 0.69 | 0.47 |
| Adiponectin (ng/ml) | 7,032 (4,243;11,470) | 6,923 (4,101;12,080) | 7,064 (4,142;12,020) | 0.97 | 0.77 |
| CRP (ng/ml) | 848 (359;2,032) | 829 (378;1,921) | 871 (383;2,124) | 0.46 | 0.92 |
| **Fasting serum lipids** | | | | | |
| Triglyceride (mmol/l) | 1.0 (0.8;1.5) | 1.1 (0.8;1.6) | 1.1 (0.8;1.6) | 0.02 | 0.07 |
| Total cholesterol (mmol/l) | 5.52 ± 1.08 | 5.56 ± 1.06 | 5.48 ± 1.07 | 0.50 | 0.34 |
| HDL-cholesterol (mmol/l) | 1.45 ± 0.40 | 1.44 ± 0.40 | 1.42 ± 0.39 | 0.03 | 0.10 |
| LDL-cholesterol (mmol/l) | 3.51 ± 0.96 | 3.58 ± 1.0 | 3.43 ± 0.94 | 0.43 | 0.28 |
| **Glucose homeostasis** | | | | | |
| Fasting plasma glucose (mmol/l) | 5.54 ± 0.76 | 5.54 ± 0.80 | 5.53 ± 0.85 | 0.99 | 0.56 |
| Fasting serum insulin (pmol/l) | 34 (24;51) | 34 (24;51) | 35 (24;53) | 0.41 | 0.90 |
| HOMA-IR (mmol/lxpmol/l) | 8.4 (5.7;12.9) | 8.2 (5.6;12.9) | 8.4 (5.7;13.1) | 0.42 | 0.85 |
| Insulinogenic index (pmolxpmol^-1^) | 28.48 ± 18.98 | 29.55 ± 19.59 | 30.06 ± 20.66 | 0.04 | 0.09 |
| ***PTER*** | **CC** | **CT** | **TT** | ***p*_add_** | ^a^***p*_add_** |
| **rs10508503** |  |  |  |  |  |
| N | 4,895 | 901 | 49 |  |  |
| (men/women) | (2,457/2,438) | (439/462) | (21/28) |  |  |
| Age (years) | 46 ± 8 | 46 ± 8 | 47 ± 7 |  |  |
| **Obesity-related measures** | | | | | |
| BMI (kg/m^2^) | 26.2 ± 4.5 | 26.3 ± 4.5 | 26.2 ± 4.8 | 0.63 | - |
| Weight (kg) | 78.1 ± 16.0 | 78.4 ± 15.6 | 76.4 ± 17.7 | 0.48 | - |
| Height (cm) | 172.3 ± 9.2 | 172.4 ± 9.4 | 170.4 ± 9.5 | 0.44 | - |
| Waist (cm) | 86.5 ± 13.3 | 86.5 ± 13.0 | 85.0 ± 12.5 | 0.88 | - |
| Waist-Hip ratio | 0.86 ± 0.09 | 0.85 ± 0.08 | 0.84 ± 0.07 | 0.40 | - |
| **Fasting serum adipokines** | | | | | |
| Leptin (ng/ml) | 5.5 (2.6;11.6) | 5.8 (2.6;12.4) | 5.2 (2.6;13.2) | 0.66 | 0.83 |
| Adiponectin (ng/ml) | 6,980 (4,202;11,930) | 7,152 (4,117;12,470) | 6,232 (2,742;12,910) | 0.89 | 0.99 |
| CRP (ng/ml) | 847 (378;2,009) | 837 (357;1,887) | 1,036 (445;2,225) | 0.49 | 0.39 |
| **Fasting serum lipids** | | | | | |
| Triglyceride (mmol/l) | 1.1 (0.8;1.5) | 1.1 (0.8;1.5) | 1.1 (0.8;1.6) | 0.66 | 0.79 |
| Total cholesterol (mmol/l) | 5.52 ± 1.07 | 5.56 ± 1.07 | 5.6 ± 0.95 | 0.36 | 0.38 |
| HDL-cholesterol (mmol/l) | 1.43 ± 0.40 | 1.45 ± 0.40 | 1.56 ± 0.48 | 0.19 | 0.11 |
| LDL-cholesterol (mmol/l) | 3.52 ± 0.98 | 3.55 ± 0.95 | 3.56 ± 0.87 | 0.72 | 0.77 |
| **Glucose homeostasis** | | | | | |
| Fasting plasma glucose (mmol/l) | 5.54 ± 0.80 | 5.51 ± 0.66 | 5.6 ± 0.75 | 0.64 | 0.50 |
| Fasting serum insulin (pmol/l) | 34 (24;51) | 35 (24;50) | 36 (25;49) | 0.24 | 0.33 |
| HOMA-IR (mmol/lxpmol/l) | 8.3 (5.6;12.9) | 8.4 (5.7;12.9) | 8.8 (5.7;12.8) | 0.34 | 0.48 |
| Insulinogenic index (pmolxpmol^-1^) | 29.51 ± 19.97 | 28.69 ± 18.04 | 27.35 ± 15.68 | 0.42 | 0.30 |

Data are presented as mean ± SD when following a normal distribution. Remaining traits are presented as median (inter-quartile range). Multiple regression analysis was used to test for differences between genotype groups. *P*-values are adjusted for age and sex assuming an additive model. ^a^*P*-values additionally adjusted for BMI. Homeostasis model assessment of insulin resistance (HOMA-IR) was calculated as ((fasting plasma glucose (mmol/l) x fasting serum insulin (pmol/l)) / 22.5). Insulinogenic index was calculated as ((serum insulin 30 minuttes post-OGTT (pmol/l) – fasting serum insulin (pmol/l) / plasma glucose 30 minuttes post-OGTT (mmol/l)). LDL-cholesterol was calculated as. ((total cholesterol (mmol/l) – HDL-cholesterol (mmol/l) – triglyceride (mmol/l) / 2.2).
