# Supplementary material for: Studies of Metabolic Phenotypic Correlates of 15 Obesity Associated Gene Variants
Source: PLoS One. 2011 Sep 2;6(9):e23531. doi: 10.1371/journal.pone.0023531 (PMC3166286; doi:10.1371/journal.pone.0023531)
Supplement: Note S1 — Individuals included from the Inter99 cohort. (DOCX) [file pone.0023531.s006.docx]

**Note S1. Individuals included from the Inter99 cohort.**

5,586 individuals from the Inter99 cohort were included in the follow-up of the top 43 variants in the BMI GWAS performed by Thorleifsson *et al.* and for additional three variants 5,450 individuals from the ADDITION study were included. Furthermore, association studies with obesity for the BMI- and weight-associated variants were performed in a study sample comprising 22,277 individuals of mixed ethnicity of which 3,362 individuals were included in the present study. A total of 4,841 controls from the Inter99 study and 2,471 cases from the Steno outpatient clinic were additionally included in the association study of type 2 diabetes.
