# Supplementary material for: Studies of Metabolic Phenotypic Correlates of 15 Obesity Associated Gene Variants
Source: PLoS One. 2011 Sep 2;6(9):e23531. doi: 10.1371/journal.pone.0023531 (PMC3166286; doi:10.1371/journal.pone.0023531)
Supplement: Note S2 — SNPs selected for genotyping. (DOCX) [file pone.0023531.s007.docx]

**Note S2.** **SNPs selected for genotyping.**

Four SNPs were identified in the *TMEM18* locus, and the association signal was captured by rs7561317 (r^2^>0.85) which lies 23 kb downstream of *TMEM18*. Three SNPs were identified in the *SH2B1* locus, with rs7498665 located in exon 5 (Ala484Thr) overlapping between the two GWAS, and capturing the association signal (r^2^>0.96). Two SNPs were identified in the *KCTD15* locus, with rs29441 located 4.4 kb downstream of *KCTD15*, being an approvable proxy for both SNPs (r^2^=0.64). Three SNPs were identified in the *NEGR1* locus, with rs2568958 16.8 kb upstream of *NEGR1* being a perfect proxy for all three SNPs (r^2^=1.0). The *BDNF* locus represented by five SNPs was only identified in one GWAS, however, this association signal is captured by rs4923461 and rs9259946 (r^2^>0.84). As for the remaining loci only one SNP in each loci was identified; *SEC16B* rs10913469, *ETV5 rs*7647305, *FAIM2* rs7138803, *GNPDA2* rs10938397, *MTCH2* rs10838738, *BAT2* rs2260000, *NPC1* rs1805081, *MAF* rs1424233, and *PTER* rs10508503, and these were genotyped.
